# Interferon-gamma signaling promotes cartilage regeneration after injury

**DOI:** 10.1038/s41598-024-58779-0

**Published:** 2024-04-05

**Authors:** Ju-Ryoung Kim, Bong-Ki Hong, Thi Hong Nhung Pham, Wan-Uk Kim, Hyun Ah Kim

**Affiliations:** 1 Division of Rheumatology, Department of Internal Medicine, Hallym University Sacred Heart Hospital, 896, Pyungchon, Anyang, Kyunggi 14068 Korea; 2 Institute for Skeletal Aging, Hallym University, Chuncheon, Gangwon-do 24252 Korea; 3 Division of Rheumatology, Department of Internal Medicine, School of Medicine, The Catholic University of Korea, Seoul, 06591 Korea; 4 Center for Intergrative Rheumatoid Transcriptomics and Dynamics, School of Medicine, The Catholic University of Korea, Seoul, 06591 Korea

**Keywords:** Cell biology, Molecular biology

## Abstract

Osteoarthritis is a common chronic disease and major cause of disability and chronic pain in ageing populations. In this pathology, the entire joint is involved, and the regeneration of articular cartilage still remains one of the main challenges. Here, we investigated the molecular mechanisms underlying cartilage regeneration in young mice using a full-thickness cartilage injury (FTCI) model. FTCI-induced cartilage defects were created in the femoral trochlea of young and adult C57BL/6 mice. To identify key molecules and pathways involved in the early response to cartilage injury, we performed RNA sequencing (RNA-seq) analysis of cartilage RNA at 3 days after injury. Young mice showed superior cartilage regeneration compared to adult mice after cartilage injury. RNA-seq analysis revealed significant upregulation of genes associated with the immune response, particularly in the IFN-γ signaling pathway and qRT-PCR analysis showed macrophage polarization in the early phase of cartilage regeneration (3 days) in young mice after injury, which might promote the removal of damaged or necrotic cells and initiate cartilage regeneration in response to injury. IFN-γR1- and IFN-γ-deficient mice exhibited impaired cartilage regeneration following cartilage injury. DMM-induced and spontaneous OA phenotypes were exacerbated in IFN-γR1^−/−^ mice than in wild-type mice. Our data support the hypothesis that IFN-γ signaling is necessary for cartilage regeneration, as well as for the amelioration of post-traumatic and age-induced OA.

## Introduction

Osteoarthritis (OA) imposes a significant burden on aging societies around the world. A key pathological process in OA is articular cartilage degeneration, which is difficult to treat because of the low proliferation rate of chondrocytes, as well as the low intrinsic repair capacity of adult articular cartilage due to a lack of blood vessels and nerves. Cartilage regeneration is more efficient in young animals; understanding the molecular and cellular basis for cartilage regeneration in these animals may facilitate treatment of OA and other cartilage disorders^1–3^.

Tissue regeneration is a tightly regulated process involving growth factors, cytokines, and proteinases, which act in concert to regulate cell density, proliferation, and migration, as well as the extracellular matrix (ECM) composition and inflammatory response^4–6^. A growing body of evidence indicates that inflammatory cells play an important regulatory role in cartilage regeneration after injury^7–9^. In particular, the innate immune response, which is a major event in the early stages of tissue regeneration, is a crucial determinant of the speed and outcome of the repair process. Initially, neutrophils accumulate in injured cartilage to facilitate the phagocytosis of tissue debris and production of proinflammatory cytokines, such as tumor necrosis factor (TNF)-α and interleukin (IL)-1β, which are believed to play a role in the early phase of tissue regeneration^10^. Macrophages accumulate in later phases and contribute to tissue repair via pro- and antiinflammatory mechanisms, which depend on M1 and M2 macrophage polarization, respectively^11^. Differences between young and old animals in the timing, magnitude, and characteristics of the macrophage response to cartilage injury have been reported; in particular, M2 macrophage polarization is delayed or reduced in the cartilage of old animals^12^.

Interferons (IFNs) are a family of proteins also known as an immunoregulatory cytokines. Human IFNs are classified into three types based on the receptors that they interact with and their amino acid sequences^13^. Type I interferons include 12 types of IFN-alpha (IFN-α), as well as IFN-beta (IFN-β), IFN-epsilon, IFN-kappa and IFN-omega; these IFNs play a role in viral replication and activation of innate and adaptive immune responses^14^. By contrast, there is only one type II IFN, IFN-gamma (IFN-γ), which binds to interferon gamma receptor 1 (IFNGR) 1 and IFNGR2 (high- and low-affinity receptors, respectively). IFN-γ also plays an important role in innate and adaptive immune responses by activating monocytes and macrophages^15^. Type III IFNs include four types of IFN-lambda. IFNs were initially considered anti-viral cytokines, but more recent studies have reported diverse biological functions of IFNs including roles in anti-cancer immunity, the pathogenesis of autoimmune diseases, and the regulation of pain signals and tissue regeneration^16–18^. In particular, because IFNs can regulate the inflammatory milieu at the site of injury, they may play a role in tissue repair.

In this study, we investigated the mechanisms underlying cartilage regeneration in a mouse model of cartilage injury by comparing the molecular profiles of young and old mice and found that the IFN-γ signaling pathway plays a major role in the regeneration process.

## Materials and methods

### Experimental animals

All procedures were performed in accordance with relevant guidelines and regulations. All mouse experiments and protocols were approved by the Care and Use of Laboratory Animals Committee of the National Veterinary Research & Quarantine Service of South Korea, the Hallym Medical Center Institutional Animal Care and Use Committee (HMC 2017-1-0831-25), and ARRIVE guidelines (https://arriveguidelines.org). Specific pathogen-free 3-week-old or 10-week-old C57BL/6 mice were purchased from Dooyeol Biotech (Seoul, Korea). IFNGR1^flux/flux^ mice were purchased from Jackson Laboratory (Jax 025394, USA). Col2a-cre mice were generously provided by Professor Jang-Soo Chun (Gwangju Institute of Science and Technology, Korea). IFN-γ^−/−^ mice were generously provided by Professor Mi-La Cho (Catholic University, Korea). Most mice (IFNGR1^flux/flux^, Col2a-cre and IFN-γ^−/−^ mice) used in our experiments were analyzed on C57BL/6 background. The mice cages were maintained in temperature controlled (23 ± 2.0 °C) and humidity controlled (50 ± 10%) environment with 12 air changes hourly and an alternating 12 h cycle of light and dark and were provided with mouse nest (Diamond Nest, USA) for enrichment. Three or four mice were housed per a polycarbonate cage (380 × 230 × 195 mm^3^) with replaceable filter paper lid containing autoclaved woodchip and pulpchip bedding with free access to food (Teklad Global 18% protein rodent diet, Envivo) and autoclaved tap water^19^.

### Full-thickness cartilage injury (FTCI) animal model

FTCI surgery of the left-knee was performed in 3-week old or 10-week old male C57BL/6 mice, or 3-week old IFN-γR1^−/−^ or IFN-γ^−/−^ mice. FTCI was conducted using a modified version of the previously established protocol^2^. Briefly, local anesthetic solution was prepared as follows. Zoletil 50 (a mixture of 1:1 tiletamine/zolazepam, Virbac, Carros, France) powder was reconstructed at a concentration of 50 mg/mL by addition of 5 mL of autoclaved water and the stock mixture was diluted with Rompun (xylazine hydrochloride, 23.32 mg/mL, Bayer, Leverkusen, Germany) and phosphate-buffered saline (PBS pH 7.4) at 1:4:10 ratios. Mice were anaesthetized by intraperitoneal injection of an anesthetic solution (40 μL/mouse for 3-week-old and 120 μL/mouse for 10-week-old). A longitudinal full thickness injury was made in the patellar groove of the femur on the left knee using a custom made device in which tape in blue color was placed approximately 300 μm to the tip of 26-G needle. After creating the injury, the articular capsule and the skin were independently closed with 6-0 nylon surgical sutures (Ailee, Busan, South Korea). Mice were sacrificed at the designated time points after surgery (Supplementary Fig. 1A).

### OA induction

DMM (destabilization of the medial meniscus) surgery of the right knee was performed in 10-week old male WT (C57BL/6 background) and IFN-γR1^−/−^ mice^20^. Briefly, mice were anaesthetized by intraperitoneal injection of an anesthetic mixture as described above. The joint capsule medial to the patellar tendon was then incised with scissors, and the meniscotibial ligament of the medial meniscus was transected. For sham surgery, the meniscotibial ligament was exposed but the ligament was left intact. The joint capsule and skin were closed using 6-0 blue nylon surgical sutures. Mice were sacrificed at 12 weeks after surgery.

### Spontaneous OA in aged animal

During breeding and experiments, WT (n = 7) and IFN-γR1^−/−^ (n = 7) mice were housed in sterilized microbarrier unit under SPF conditions. These mice received autoclaved food and water as stated earlier. The mice were sacrificed by cervical dislocation at the age of 16 month. In all cases, the left knee joint was removed.

### Histologic evaluation using the cartilage repair, Osteoarthritis Research Society International (OARSI), synovitis, subchondral bone, and osteophyte scoring systems

For an evaluation of the cartilage repair, paraffin blocks were sectioned at three points per knee joint. The first section (A) was 100 mm proximal to the intercondylar notch, the second (B) 100 mm proximal to the section A, and the third (C) was 100 mm proximal to the section B. For histomorphometry and scoring, sections at each point, A–C, were used. For an evaluation of the cartilage degeneration, a series of three 5-µm sections taken at 200-μm intervals in the coronal plane. For safranin-O (0.1%) and fast green staining and hematoxylin sections were deparaffinized in xylene and hydrated by using a graded ethanol series. To assess cartilage regeneration in FTCI mouse model, safranin-O stained samples were graded based on the previous reported histological repair scoring system^2^. To evaluate the extent of cartilage destruction in DMM mouse model, safranin-O stained samples were graded according to the Osteoarthritis Research Society International (OARSI) scoring^21^ and subchondral bone sclerosis, osteophytes, and synovitis were also scored by blinded observers. Details of each scoring system are provided in the Supplementary data. All histology specimens were imaged on a Nikon ECLIPSE microscope equipped with a digital camera (Nikon DS-Fi2).

### Immunohistochemistry and immunofluorescence

Sections were deparaffinized in xylene and hydrated by using a graded ethanol series. For immunohistochemistry, the following primary antibodies were used: anti-type II collagen (Abcam, ab34712, dilution 1:200), anti-aggrecan (Millipore, AB1031, dilution 1:200), and anti-matrix metalloproteinase (MMP)-13 (Abcam, ab39012, dilution 1:200). The secondary antibody was goat anti-rabbit IgG (Santa Cruz, sc-2004, dilution 1:200). Positive results were visualized using 3,3′-diaminobenzidine (DAB) substrate staining (Vector Laboratories, SK-4100). For immunofluorescence, the following primary antibodies were used: anti-type II collagen (Abcam, ab34712, dilution 1:500), anti-aggrecan (Millipore, AB1031, dilution 1:400), and anti-IFN-γR1 (GeneTex, GTX103098, dilution 1:500). The secondary antibody was Alexa Fluor 555 goat anti-rabbit IgG (H + L, Invitrogen, A21428).

### RNA preparation and RNA-seq analysis

For analysis of mRNA expression, cartilages were dissected, and tissues were rapidly frozen in liquid N_2_. Total RNA was isolated from pools of at least 10 cartilages from 10 injured mice or 5 control, uninjured mice by 500 μL of Trizol (Invitrogene, 15596018) using a polytron. RNA-seq library preparation and sequencing was performed in accordance with a previously reported method^22^. Total RNA concentration was calculated by Quant-IT RiboGreen (Invitrogen, #R11490). To assess the integrity of the total RNA, samples are run on the *TapeStation RNA screentape (Agilent, #5067-5576).* Only high-quality RNA preparations, with RNA integrity number greater than 7.0, were used for RNA library construction. A library was independently prepared with 1 μg of total RNA for each sample by Illumina TruSeq Stranded mRNA Sample Prep Kit (Illumina, Inc., San Diego, CA, USA, #RS-122-2101). The first step in the workflow involves purifying the poly‐A containing mRNA molecules using poly‐T‐attached magnetic beads. Following purification, the mRNA is fragmented into small pieces using divalent cations under elevated temperature. The cleaved RNA fragments are copied into first strand cDNA using SuperScript II reverse transcriptase (Invitrogen, #18064014) and random primers. This is followed by second strand cDNA synthesis using DNA Polymerase I, RNase H and dUTP. These cDNA fragments then go through an end repair process, the addition of a single ‘A’ base, and then ligation of the adapters. The products are then purified and enriched with PCR to create the final cDNA library. The libraries were quantified using KAPA Library Quantification kits for Illumina Sequencing platforms according to the qPCR Quantification Protocol Guide (KAPA BIOSYSTEMS, #KK4854) and qualified using the TapeStation D1000 ScreenTape (Agilent Technologies, # 5067-5582). Indexed libraries were then submitted to an Illumina NovaSeq (Illumina, Inc., San Diego, CA, USA), and the paired-end (2 × 100 bp) sequencing was performed.

### RNA-seq data collection

FASTQ files and FPKM data were entered into the NCBI Gene Expression Omnibus (https://www.ncbi.nlm.nih.gov/geo/). Accession and data citation numbers were assigned GSE232013.

### 5-ethynyl-2′-deoxyuridine (EdU) proliferation assays

To determine the proliferative capacity of the cells, we injected WT (n = 7) or IFN-γR1^−/−^ (n = 7) mice with 0.1 mg/20 g body weight of EdU 2 h prior to tissue collection on day7 after FTCI. After tissue preparation, we used a Click-iT reaction kit (Invitrogen C10640) as per manufacturer’s guidelines using a fluorescence Alexa Fluor 555 or 647 to detect the incorporated EdU. In cultured cells grown on chamber slides (Sigma C7182), EdU was added to a concentration of 10 mM for 45 min and detected via the Click-iT reaction Kit following IF procedures.

### Scratch wound healing assay and transwell migration assay

Cell scratch and migration assays were conducted using the previously reported method^23^. Briefly, for the scratch assay, ATDC5 cells, a chondrogenic progenitor cell line, were transfected with IFN-γR1 siRNA or siControl after seeding at approximately 80% confluency onto 6-well plates, and incubated at 37 °C. After 24 h of transfection, a vertical scratch wound was made through the center of each well using a 100-µL pipette tip and then the cell were washed three times with PBS to remove the scratched cells, and fresh serum-free medium with IFN-γ (2 μg/mL as final concentration) was transferred. After 24 h the cells were examined by light microscopy at a magnification of × 100 to determine the resealing of the cell monolayer. For Transwell migration assay, after transfection with IFN-γR1 siRNA or siControl, ATDC5 cells were trypsinized and placed into the upper wells of the Boyden chamber (100,000 cells/well) in 100 µL DMEM with 20% FBS and IFN-γ. In the lower chamber, 600 µL of DMEM containing 10% FBS was added. Cells in the Boyden chamber were incubated for 48 h at 37 °C in a 5% CO_2_ incubator. After non-migrated cells were scraped off, the membrane was fixed with methanol, stained with Hemacolor stain set (EM Industries, Inc, Gibbstown, NJ). Relative migration was based on the average number of cells on the underside of the membrane in four random images generated at 20 × magnification under microscope.

### Patients

Human cartilage samples were obtained at the time of total knee replacement surgery from OA patients (n = 18, mean ± standard deviation age 71.4 ± 6.3 years) who were diagnosed according to the American College of Rheumatology criteria^24^. Normal cartilage samples were obtained from the femoral head of patients (n = 9, 72.6 ± 8.5 years) with femoral neck fractures with no known history of OA or RA. The collection and use of human tissue samples were reviewed and approved by the Institutional Review Board of Hallym University Sacred Heart Hospital, Anyang, Korea (approval number 2013-I022)^25^. All patients provided written informed consent for the use of the discarded cartilage samples. Research involving human research participants have been performed in accordance with the Declaration of Helsinki.

### Cell culture

We isolated primary chondrocytes from the articular cartilage as previously described^26^. Briefly, articular cartilage was digested using protease (Sigma-Aldrich), collagenase (Sigma-Aldrich), and hyaluronidase from bovine testes (Sigma-Aldrich) in Dulbecco’s modified Eagle’s medium (DMEM, Life Technologies, USA). Chondrocytes were maintained in DMEM containing 10% of FBS and 1% penicillin–streptomycin. The cells were incubated at 37 °C in a humidified atmosphere of 5% CO_2_, and the medium was changed every 2–3 days. First-passage cultured human chondrocytes were used for all experiments within 3–6 days after seeding.

### Quantitative RT-PCR

Total RNA was extracted and purified from cartilage tissue, using TRIzol reagent (Invitrogene, 15596018). cDNA was synthesized from 1 µg of RNA using M-MLV reverse transcriptase (Promega, M170B). qRT-PCR analysis was performed with a QuantiFast SYBR Green PCR Kit 2000 containing cDNA, primers, and SYBR Green PCR master mix (Qiagen, 204156) and data was acquired under condition of two-step cycling, such as denaturation (95 °C, 10 s) and combined annealing/extension (58 °C, 30 s), for 45 cycles using a StepOnePlus real-time PCR system (Applied Biosystems, Waltham, MA, USA). GAPDH was used as an internal control. Primer sequences are shown in Supplementary Table S1.

### Measurement of pain-related behaviors in mice

Mechanical allodynia was assessed every 2 weeks using von Frey filaments, in accordance with a previously reported method^19^. Briefly, each mouse was placed in a dark glass on a wire mesh surface and allowed to adapt to the testing environment for 5 min. Multiple von Frey filaments with ascending gauges of stiffness (North Coast Medical, Morgan Hill, CA, USA) were then perpendicularly applied to the mouse’s hind paw until the filament bent. The 0.4-g filament was applied five times as the initial force. If the rate of a response, such as a brisk withdrawal or paw flinching, was < 50% (0–2 of 5 applications), the next thicker filament was tested. If the withdrawal response rate was > 50%, the test was stopped and the force of the last von Frey filament was regarded as the mechanical withdrawal threshold. The test was repeated twice for each mouse. Additionally, the withdrawal threshold in response to direct pressure on the medial part of the left knee was measured every 2 weeks using a pressure application measurement device (Ugo Basile, France, PAM-38500) as previously described with some modifications^27^. While a mouse was lightly but securely held, the sensor tip (5-mm diameter) of an applied force gauge was placed on the mouse’s knee joint. An increasingly forceful squeeze was then slowly applied across the joint at a rate of 50 g/s, with a maximum test duration of 30 s. The test was stopped when the mouse showed any behavioral signs of discomfort or distress, such as wriggling. The peak gram force (gf) applied immediately prior to discomfort sign was recorded as the base unit and designated as the limb withdrawal threshold. A cut-off force of 500 g was used to prevent trauma to the joint. The test was performed three times, with 10-min intervals, and the mean value was recorded as the nociceptive threshold.

### Statistical analysis

GraphPad Prism 8 software was used for statistical analysis. The normality of the data was determined using the Shapiro–Wilk test. Parametric t tests and Mann–Whitney U test was used for comparisons between two groups with normally and non-normally distributed data, respectively. For comparisons of > 2 conditions, one-way analysis of variance (ANOVA) or the Kruskal–Wallis test was used (depending on data distribution normality), followed by Tukey’s multiple comparisons test or Dunn’s multiple comparisons test, respectively. Pain behavior data did not follow normal distribution and was analyzed using the Kruskal–Wallis test with Dunn’s multiple comparisons test at each time point. All data are expressed as means ± standard errors of the mean (SEMs).

### Determination of sample size

Sample size for each experiment was determined using G power program (https://clincalc.com/stats/samplesize.aspx). For the capacity of cartilage regeneration by FTCI surgery we hypothesized mean articular cartilage repair scores in male mice to be 9 (SD 1.78) for the young mice group vs. 5.3 (SD 1.86) in the adult mice group. Given 80% power and an alpha level of 0.05, the power calculation suggested 4 male mice per group. In the same manner as cartilage repair score, sample sizes for DMM surgery or pain behavior measurement were calculated.

## Results

### Cartilage regeneration is enhanced in young mice compared to adult mice

**Figure 1:**
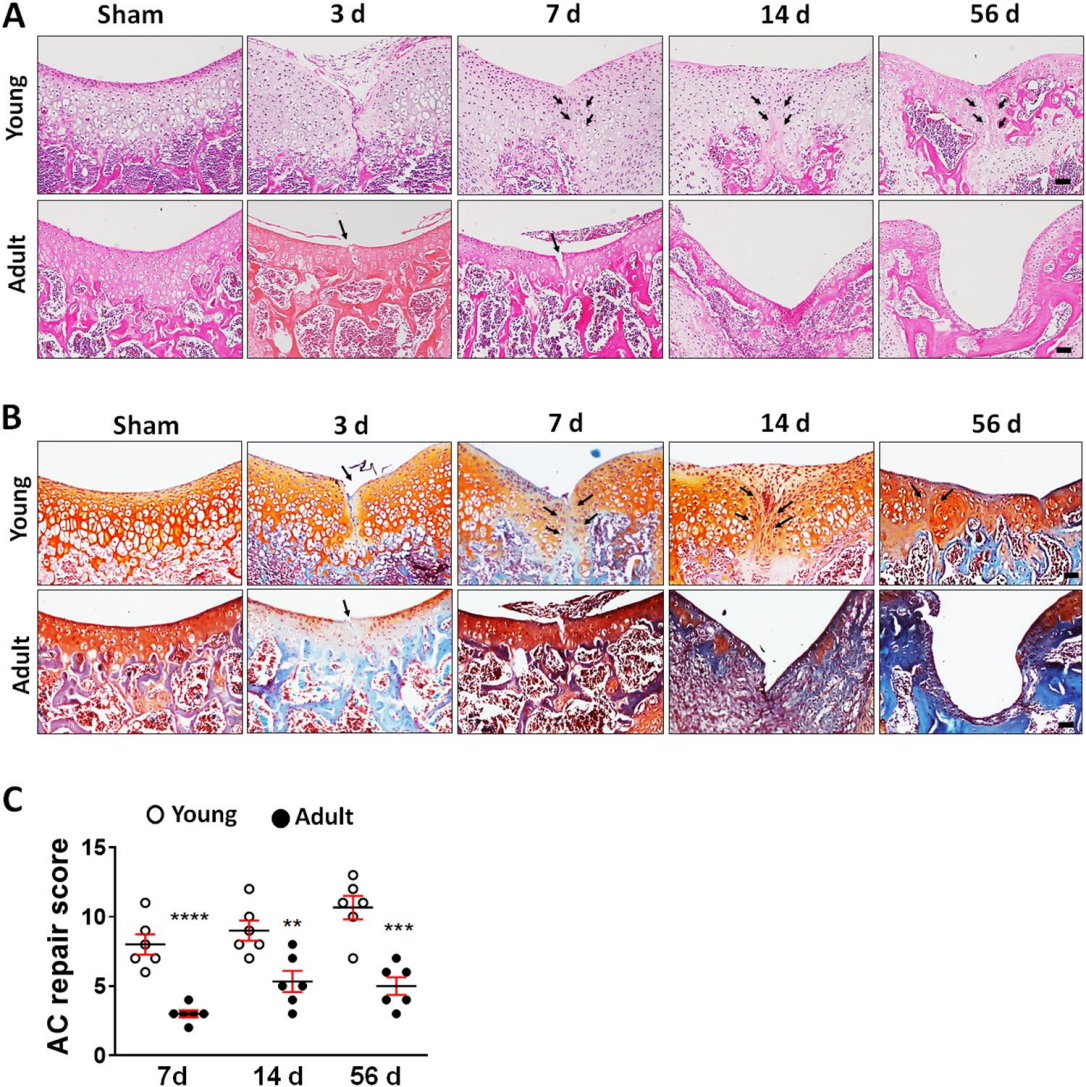
Articular cartilage repair of young and adult mice after full-thickness cartilage injury (FTCI). (**A**,**B**) Representative hematoxylin and eosin- and safranin-O/fast green- stained sections of cartilage from young (upper panels) and adult mice (lower panels) at 3, 7, 14, and 56 days post-sham operation or FTCI. Arrows indicate sites of cartilage injury. Scale bar = 50 μm. (**C**) Cartilage regeneration was evaluated using a histological scoring system at the indicated time points. Three sections from each mouse were examined and the scores were averaged. Each dot represents one mouse. *P < 0.01, **P < 0.001, ***P < 0.0001 versus the young group at each time point. (unpaired two-tailed *t*-test).

To assess how age affects articular cartilage regeneration, we induced full-thickness cartilage injury (FTCI) in the patella groove of the left knee of C57/BL6 mice (aged 3 or 10 weeks) with a 26-G needle (Supplementary Fig. 1). Histologically, the articular cartilage of young mice is thicker, with larger chondrocytes and lacunae, and the ECM shows less dense staining of proteoglycan than that of adult mice (Fig. 1A,B). In young mice, the injured area was filled with cells from 7 days after injury, with proteoglycan matrix also appearing in the defect area. Most young mice showed robust regeneration of hyaline cartilage at 14 and 56 days after injury, whereas the defect was not filled with cartilage at any point during the study period and total collapse of the joint appeared in some adult mice (Fig. 1B), such that the repair scores were higher in young than adult mice at all time points (Fig. 1C).

### Post-injury changes in cartilage anabolic markers

**Figure 2:**
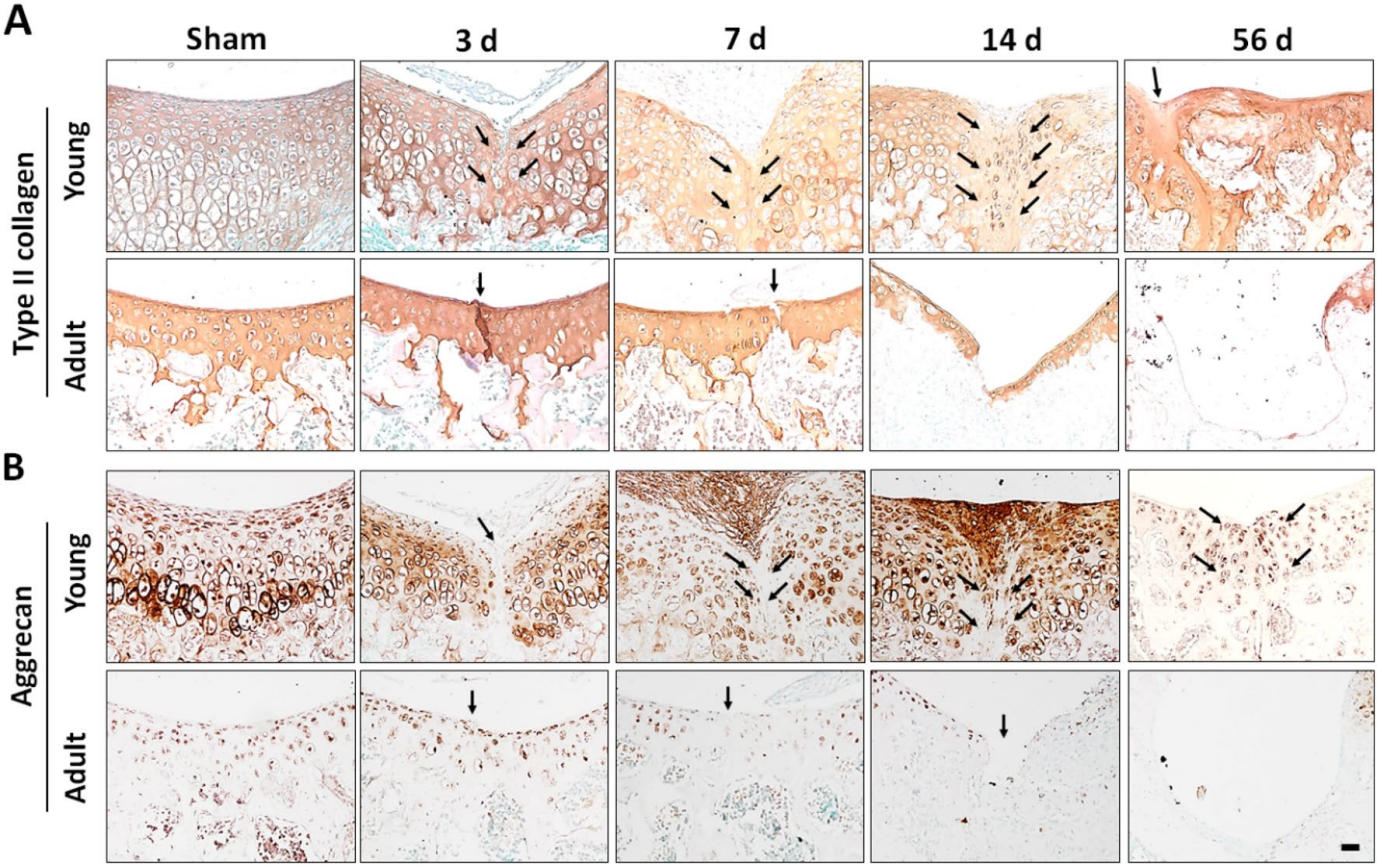
Expression of type II collagen (**A**) and aggrecan (**B**) during cartilage regeneration. Representative images of immunohistochemical staining of young (upper panel) and adult mice (lower panel) at the indicated time points post-sham operation or FTCI. Arrows indicate sites of cartilage injury.

The expression of aggrecan and Sox9, which are cartilage anabolic genes, in response to injury was examined in young and old mice. The mRNA expression of aggrecan and Sox9 in young mice decreased at 3 days post injury but subsequently started to increase, peaking at 14 days (Supplementary Fig. 2). By contrast, significant changes in the expression of aggrecan and Sox9 were not seen in adult mice throughout the study period (Supplementary Fig. 2). Young mice showed robust staining for type II collagen and aggrecan at the site of injury, whereas adult mice showed little or no staining at any time point (Fig. 2A,B). These results indicate that anabolic responses to cartilage injury are more dynamic in young compared to adult mice.

### Cartilage injury upregulates IFN-related genes in young mice

**Figure 3:**
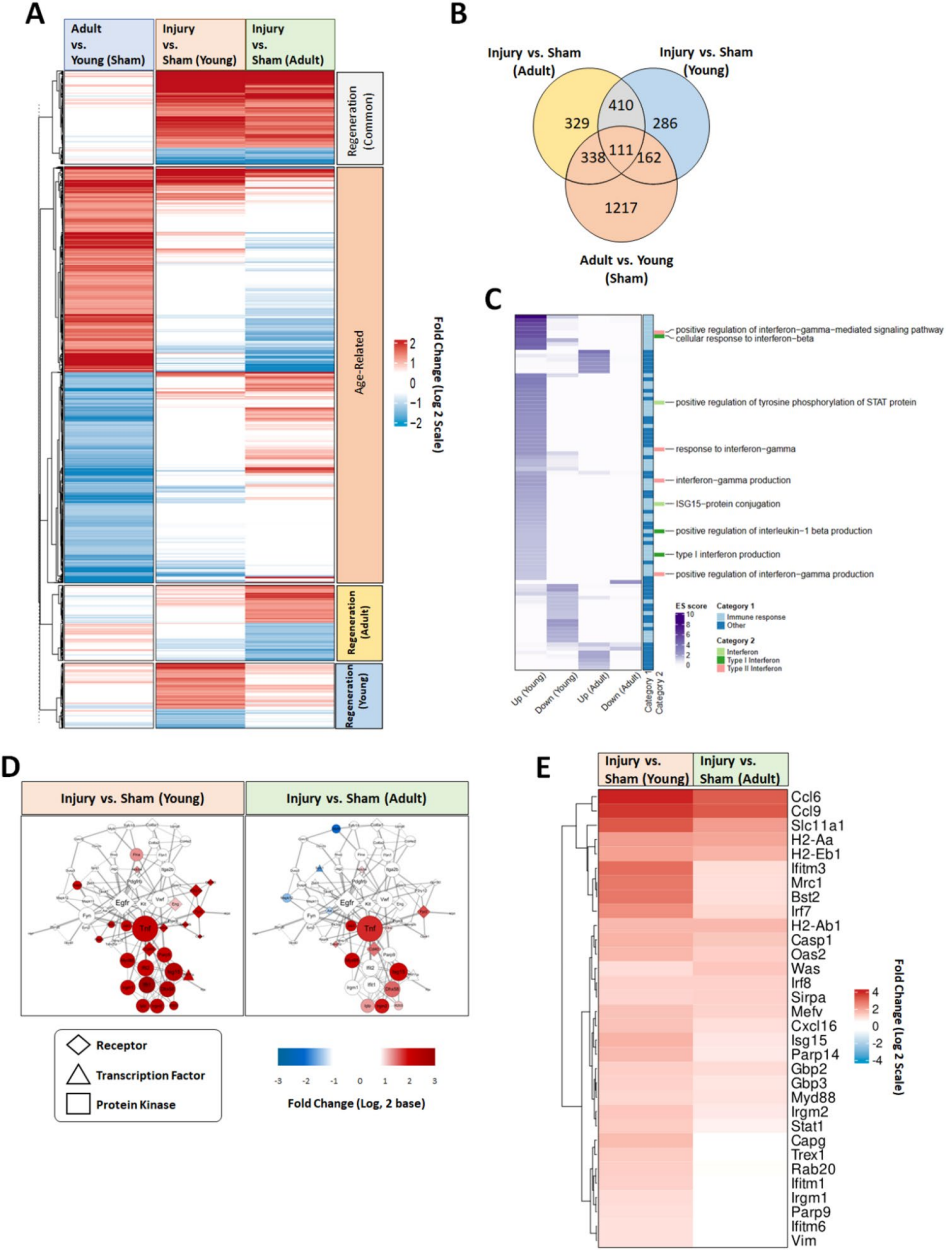
Overview of differentially expressed genes (DEGs) between young and adult mice after FTCI. (**A**) Heat map of 2853 DEGs grouped by cartilage regeneration (blue = decreased expression; red = increased expression) and age. (**B**) Venn diagram showing overlapping DEGs in three group comparisons: adult versus young mice, young injured versus young sham mice, and adult injured versus adult sham mice. The numbers of DEGs are shown indicated in each section of the Venn diagram; the same color scheme is used for the DEG clusters in the heatmap. (**C**) Heatmap showing Gene Ontology biological processes (GOBPs) enriched by upregulated or downregulated DEGs in chondrocytes from injured and sham group cartilage. The color gradient represents the enrichment score (in − log^10^ units) for each GOBP. (**D**) Network model of immune response-related genes involved in the cartilage regeneration process. Node colors reflect fold-change values in the injured versus sham control comparison (red = upregulation, blue = downregulation). (**E**) Heatmap of DEGs related to interferon-gamma (IFN-γ) response genes and fold-change values.

To identify the key molecules and pathways involved in the regeneration of cartilage in young mice, we performed RNA sequencing (RNA-seq) analysis (Fig. 3). Total RNA was isolated from pooled sham group cartilage, and from cartilage at 3 days after FTCI, from young (3 weeks old; n = 15) and adult (10 weeks old; n = 15) C57BL/6 mice, respectively. We identified 2853 differentially expressed genes (DEGs) and divided them into four major clusters: age-related, regeneration-related in both young and adult mice, regeneration-related only in young mice (“Young-up”), and regeneration-related only in adult mice (Fig. 3A,B). To investigate the unique biological processes in each cluster of DEGs, we performed a functional enrichment analysis of the DEGs in young and adult mice after injury. Genes involved in immune response-related biological processes, particularly IFN signaling (including positive regulation of the IFN-γ-mediated signaling pathway and the cellular response to IFN-β) were among the most significantly upregulated in the Young-up cluster. By contrast, genes involved in immune response-related processes in adult cartilage did not show expression changes (Fig. 3C). To investigate the relationships among cartilage regeneration-related immune response genes and compare their expression levels between young and adults, we constructed a network model of immune response-related genes based on protein–protein interaction data (Fig. 3D). In accordance with Gene Ontology data, IFN-related genes showed significant expression differences between young and adult mice during the cartilage regeneration process. We focused on IFN-γ because it had a higher enrichment score than type I IFN genes (Fig. 3C) and reportedly plays a role in regeneration of diverse tissues^28^. Heatmap analysis showed that DEGs related to IFN-γ response genes were highly upregulated in young injured mice compared to adults (Fig. 3E). We postulate that the early immune response associated with IFN-γ signaling in young mice enhances cartilage regeneration.

To verify the RNA-seq data, we performed quantitative reverse transcription-polymerase chain reaction (qRT-PCR) analysis of 14 selected IFN-γ-related genes that were upregulated in the cartilage of young compared to adult mice at 3 days after injury. The RNA samples of a different set of animals were used for this analysis. Consistent with the RNA-seq results, the transcript expression levels of Ccl6, Slc11a1, Ifitm3, Mrc1, Bst2, Ifitm6, Isg15, Stat1, Capg, Trex1, Ifitm1, Irf7, and Vim were upregulated at 3 days after injury in young compared to adult mice (Supplementary Fig. 3A). Although the expression of interferon-gamma receptor 1 (IFN-γR1) and IFN-γ is more abundant in adult than in young cartilage (Supplementary Fig. 3B), young mice showed a significantly higher fold change in the expression of IFN-γR1 and IFN-γ in cartilage at 3 days after injury compared to adult mice (Supplementary Fig. 3C). These results suggests that the IFN-γ signaling pathway is involved in cartilage regeneration in young animals.

### Generation of chondrocyte-specific IFN-γ R1 knockout (KO) mice

Given that IFN-γ-related genes were upregulated in response to injury in young mice compared to adult mice at an early stage, we examined whether IFN-γ signaling contributes to cartilage regeneration. We generated mice harboring a cartilage-specific deletion of IFN-γR1 by crossing IFN-γR1flox mice with mice expressing Cre recombinase (driven by the col2a1 promoter; Supplementary Fig. 4). IFN-γR1 expression was abolished in the cartilage of KO mice, as shown by the results of RT-PCR and immunofluorescence analyses conducted at the age of 3 weeks (Supplementary Fig. 4B,C). Notably, IFN-γR1 expression was preserved in the subchondral bone of IFN-γR1^−/−^ mice. Body weight and length were not significantly different between wild-type (WT) and IFN-γR1^−/−^ mice (males and females aged 3 weeks) (Supplementary Fig. 4D). Cartilage thickness, as well as Sox9 and aggrecan expression levels, tended to be lower, while Col2a1 mRNA or protein (Type II collagen) expression was significantly lower, in KO mice (Supplementary Fig. 4E–H). There was no phenotypic difference between male and female KO mice in terms of the response to injury or cartilage characteristics; thus, we used males only in further experiments.

### IFN-γR1 deficiency impaired cartilage regeneration

**Figure 4:**
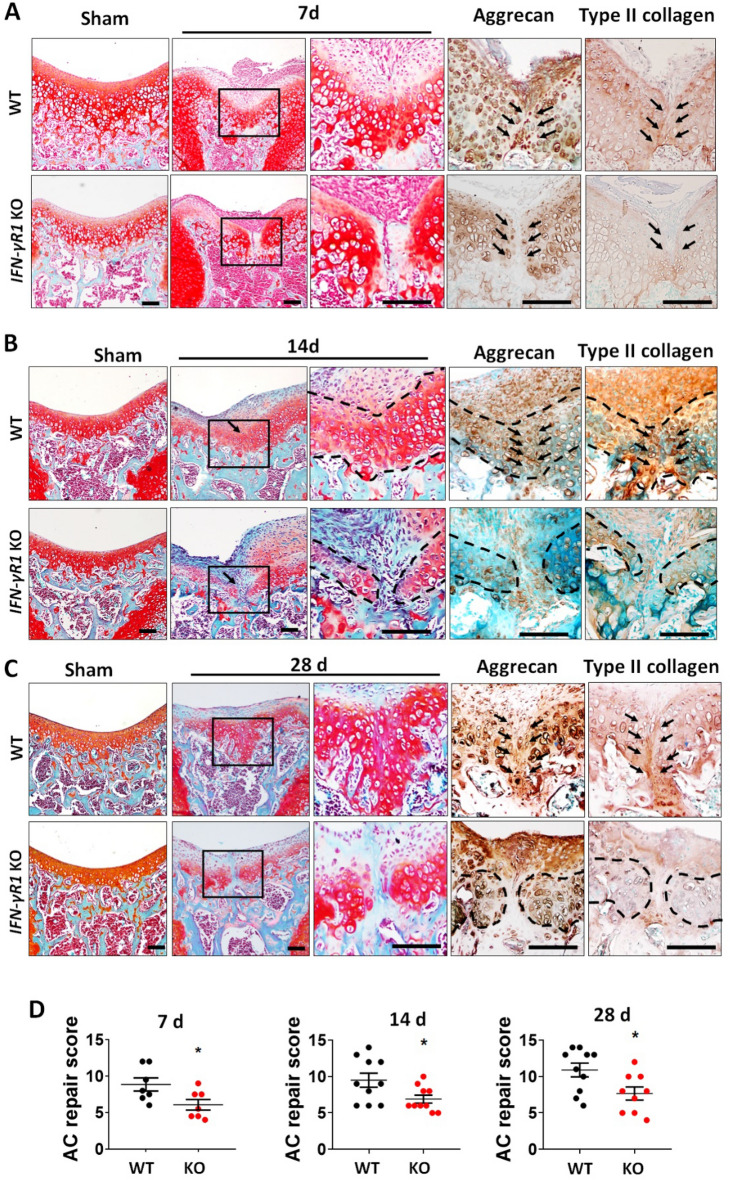
Genetic ablation of interferon-gamma receptor 1 (IFN-γR1) delayed cartilage regeneration after FTCI. (**A**–**C**) Cartilage defects and regeneration were visualized by safranin-O/fast green staining at 7 (**A**), 14 (**B**), and 28 (**C**) days after injury in 3-week-old wild-type (WT) and IFN-γR1^−/−^ mice. Boxed areas are shown at higher magnification. Representative images of immunohistochemical staining for aggrecan and type II collagen in the regenerating cartilage of WT and IFN-γR1^−/−^ mice after injury. Arrows represent injury sites. Dashed lines indicate cartilage. Scale bar = 50 μm. (**D**) Quantitative analyses of articular cartilage repair scores calculated at the indicated time points after injury for WT and IFN-γR1^−/−^ mice (n = 7–10 per group; three sections/mouse). Data are shown as mean ± standard error of the mean (SEM). *P < 0.05 versus WT controls (unpaired two-tailed *t*-test [7 days] and two-tailed Mann–Whitney U test [14 and 28 days]).

FTCI was induced in WT and IFN-γR1^−/−^ mice at 3 weeks of age, and cartilage regeneration was monitored from day 7 to day 56 after injury. After 7 days, proteoglycan matrix filling the gap induced by injury was observed in WT mice, while in IFN-γR1^−/−^ mice no cells or matrix were seen in the gap (Fig. 4A). Aggrecan and type II collagen also appeared in the defects in WT cartilage, which was not observed in the KO mice (Fig. 4A). At 14 and 28 days, WT mice showed almost complete restoration of hyaline cartilage in the injured area, whereas in IFN-γR1^−/−^ mice disruption of the ECM persisted, accompanied by a decrease in aggrecan and type II collagen expression (Fig. 4B,C). KO mice had significantly lower repair scores than WT mice at 7, 14, and 28 days post injury (Fig. 4D). At 56 days after injury, WT mice exhibited a smooth cartilage surface, normal cellularity, and robust proteoglycan staining (Supplementary Fig. 5). The gap was also filled by proteoglycan matrix in IFN-γR1^−/−^ mice, in a similar manner to WT mice at 7 days post-injury (Supplementary Fig. 5). Taken together, the results indicate that IFN-γR1 is required for cartilage repair in response to injury in young mice.

### Involvement of IFN-γ signaling in chondrocyte proliferation and migration

**Figure 5:**
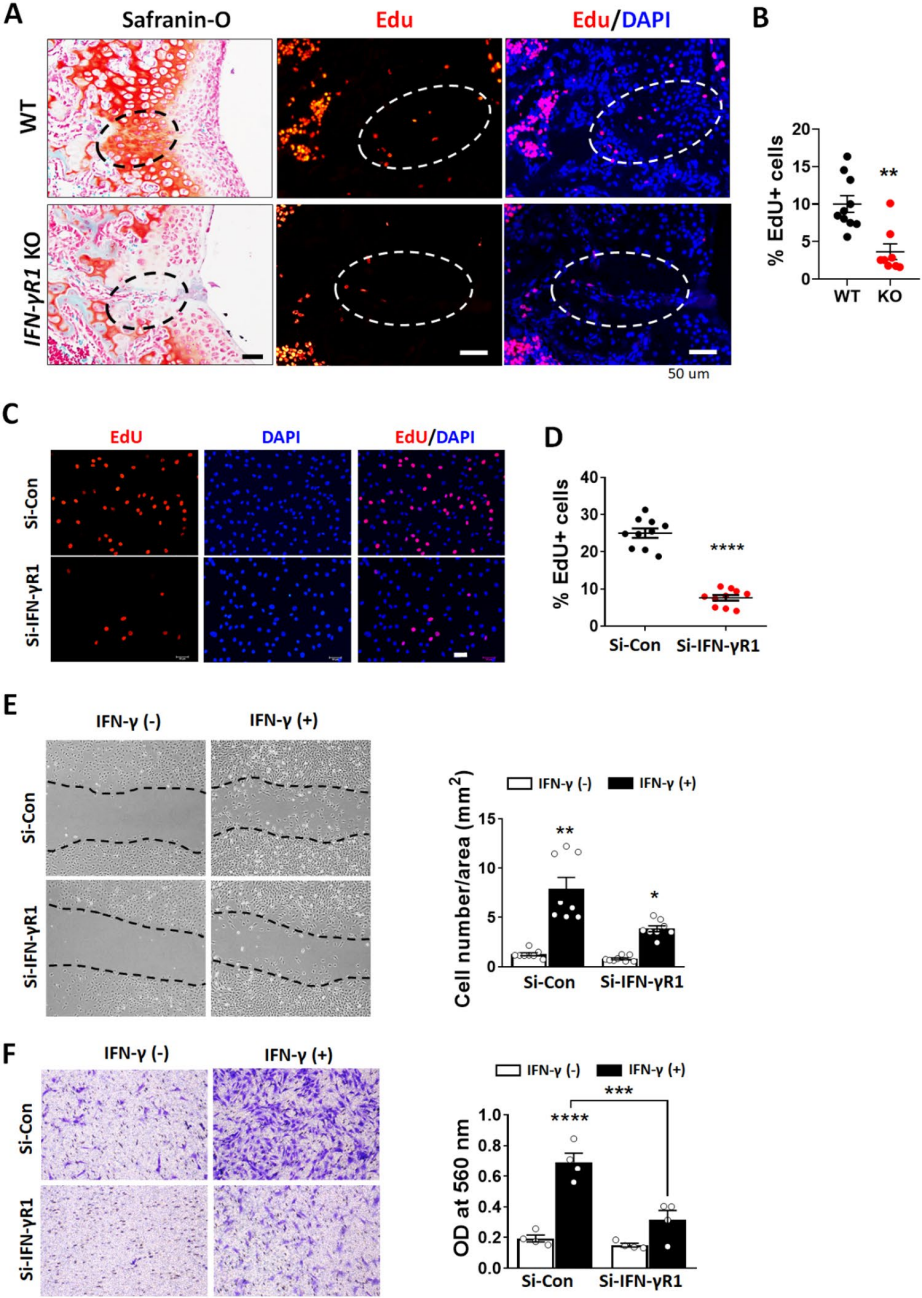
IFN-γR1 deficiency reduced cell proliferation and migration in vivo and in vitro. (**A**) Representative images of safranin-O/fast green staining of cartilage (left panels) at 7 days after injury; dashed circles indicate the injured area. Representative images of proliferating 5-ethynyl-2′-deoxyuridine (EdU)-positive cells (middle panels) and co-staining of Edu and 4′,6-diamidino-2-phenylindole (DAPI; right panel). EdU-labeled cells were detected by treating 5-µm sections with Click-iT EdU Alexa Fluor 555 reaction cocktail. Dashed circles represent the same cartilage areas shown in the left panels. Scale bar = 50 μm. (**B**) Edu-positive cells in the injured area are presented as a percentage of total cells. (**C**) Immunofluorescence analysis of EdU incorporation into si-IFN-γR1- and si-Control-transfected human chondrocytes. Representative EdU cell (red), DAPI staining (blue), and merged images (pink). Scale bar = 50 μm. (**D**) Percentages of EdU-positive human chondrocytes following transfection with si-IFN-γR1 and si-Control for 24 h. (**E**) The migration of ATDC5 cells was analyzed using scratch wound healing assay following transfection with si-IFN-γR1 or si-Control plus IFN-γ treatment for 24 h. (**F**) Results of Transwell migration assay. Images showing the migration ability of IFN-γR1 siRNA in ATDC5 cells (left panel). At 24 h after transfection with IFN-γR1 or control siRNA, ATDC5 cells were seeded into the chamber and IFN-γ was applied for 48 h to induce migration. The migrated cells were stained by crystal violet after 48 h. Absorbance data after extraction with crystal violet are shown in the right panel. Data are shown as mean ± SEM. *P < 0.05, **P < 0.01, ***P < 0.001, ****P < 0.0001 versus WT control or the siRNA control group. Statistical analyses were conducted using a two-tailed Mann–Whitney U test (**B**,**D**), Kruskal–Wallis test with Dunn’s multiple comparison test (**E**), and one-way analysis of variance (ANOVA) with Tukey’s multiple comparison test (**F**).

Successful cartilage regeneration depends on the expansion of chondrocytes and recruitment of these cells to the site of injury. To determine the role of IFN-γ signaling in the proliferation of chondrocytes, we assessed 5-ethynyl-2′-deoxyuridine (EdU) incorporation into chondrocytes after injury. At 7 days after injury, EdU was injected intramuscularly to label cells in S-phase; 2 h later, the cartilage was fixed and sectioned. We observed EdU-labeled proliferating cells in the vicinity of the defect sites in WT mice (Fig. 5 A-B), however, almost no EdU-labeled cells were detected in IFN-γR1^−/−^ mice, reflecting the significantly lower number of proliferating chondrocytes (Fig. 5A,B). Direct regulation of cell proliferation by IFN-γR1 was assessed in vitro using cultured primary human chondrocytes after knocking down IFN-γR1 (Fig. 5C,D). The number of EdU-positive cells was significantly reduced by si-IFN-γR1 compared to si-control treatment.

Because cell migration is a key process in tissue regeneration, we investigated whether IFN-γ influenced chondrocyte migration using a classical wound healing assay, Transwell migration assay, and ATDC5 cells (i.e., chondrogenic progenitor cells). IFN-γ increased migration into the scratch area which was reduced in si-IFN-γR1-transfected cells (Fig. 5E). These results were confirmed by Transwell migration assays (Fig. 5F).

### Changes in inflammatory mediators and macrophage polarization during cartilage regeneration

**Figure 6:**
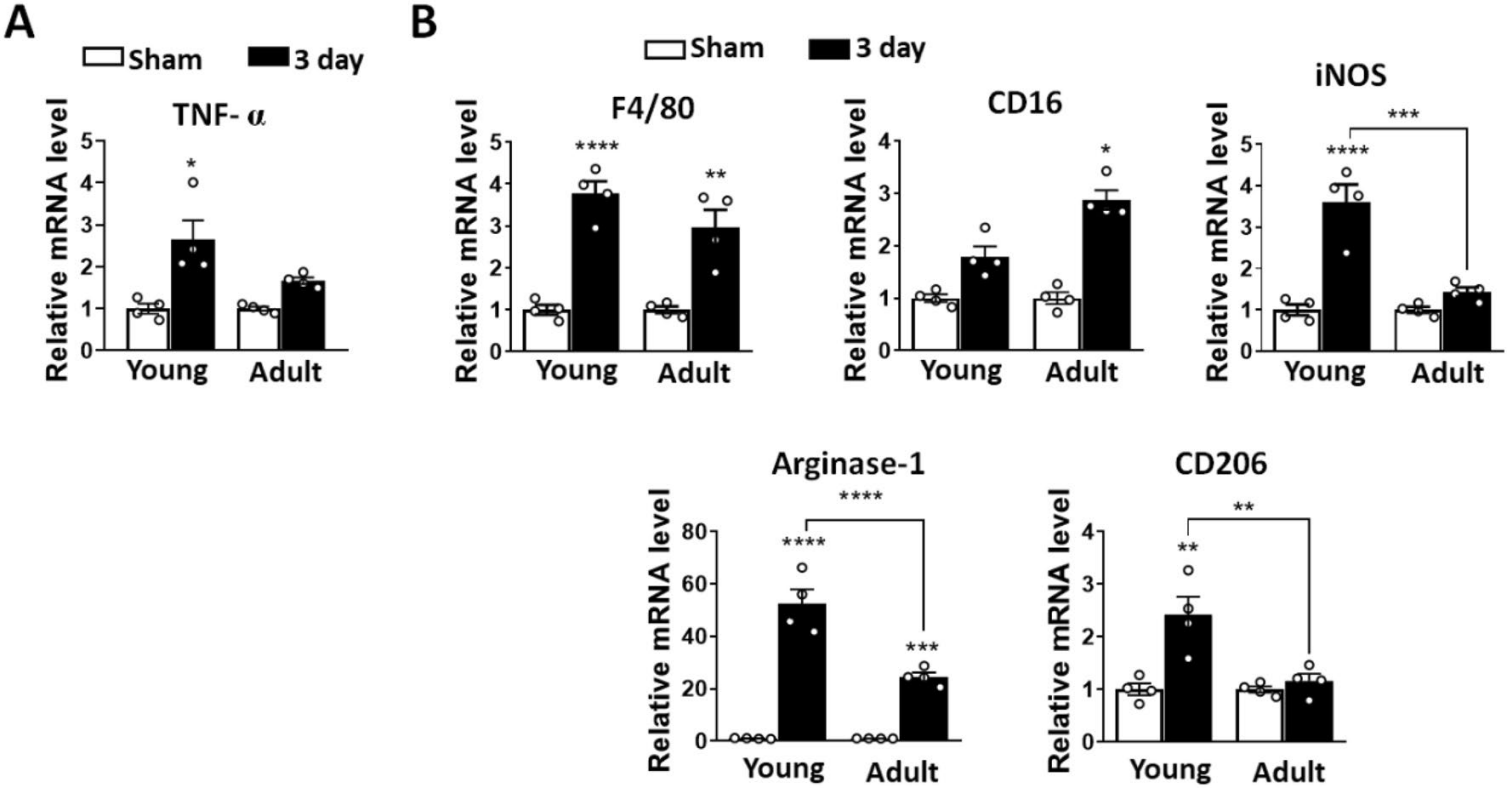
Pro-to anti-inflammatory transition of macrophage in early response following cartilage injury in young and adult mice after cartilage injury. Three-week-old (young) or ten-week-old (adult) male C57BL/6 mice were subjected to FTCI on the left knee cartilage and tissue were harvested 3 days later. The medial or lateral tibial and femoral cartilages were pooled from at least 10 mice before RNA extraction and mRNA levels were analyzed by qRT-PCR. (**A**) Expression levels of pro-inflammatory cytokine, TNF-α mRNA in the cartilage from young and adult mice. (**B**) Expression levels of common macrophage marker, F4/80, pro-inflammatory macrophage marker (M1), CD16 and iNOS and anti-inflammatory macrophage marker (M2), arginase-1 and CD206 were analyzed by qRT-PCR. Data are normalized to the expression of glyceraldehyde-3-phosphate dehydrogenase (GAPDH) mRNA and shown as means ± SEMs of duplicate experiments. *P < 0.05, **P < 0.01, ***P < 0.001, ****P < 0.0001 vs. sham control group. Statistical analyses were conducted using Kruskal–Wallis test with Dunn’s multiple comparisons test (**A**, TNF-α) (**B**, CD16, iNOS) and one-way ANOVA with Tukey’s multiple comparisons test (**B**, F4/80, Arginase-1, CD206).

In line with previous reports of roles of proinflammatory cytokines and macrophages in the early phase of cartilage regeneration^11,29–31^, we found that TNF-α was upregulated in regenerating tissue 3 days after injury in young compared to adult mice (Fig. 6A). The expression of inducible nitric oxide synthase (iNOS), which is an inflammatory marker secreted by IFN-γ, was significantly increased in young compared to adult mice, whereas the levels of CD16, which is a proinflammatory M1 marker, were decreased (Fig. 6B). In addition, young mice showed significantly elevated expression of arginase-1 and CD206, which are anti-inflammatory M2 markers compared to adult mice (Fig. 6B). Macrophage maker, F4/80 expression increased in both young and adult mice (Fig. 6B). Taken together, these data suggest that the proinflammatory response and macrophage polarization occur in the early phase of cartilage regeneration in young mice, which might promote the removal of damaged or necrotic cells and initiate cartilage regeneration in response to injury.

### IFN-γ deficiency impairs cartilage regeneration

**Figure 7:**
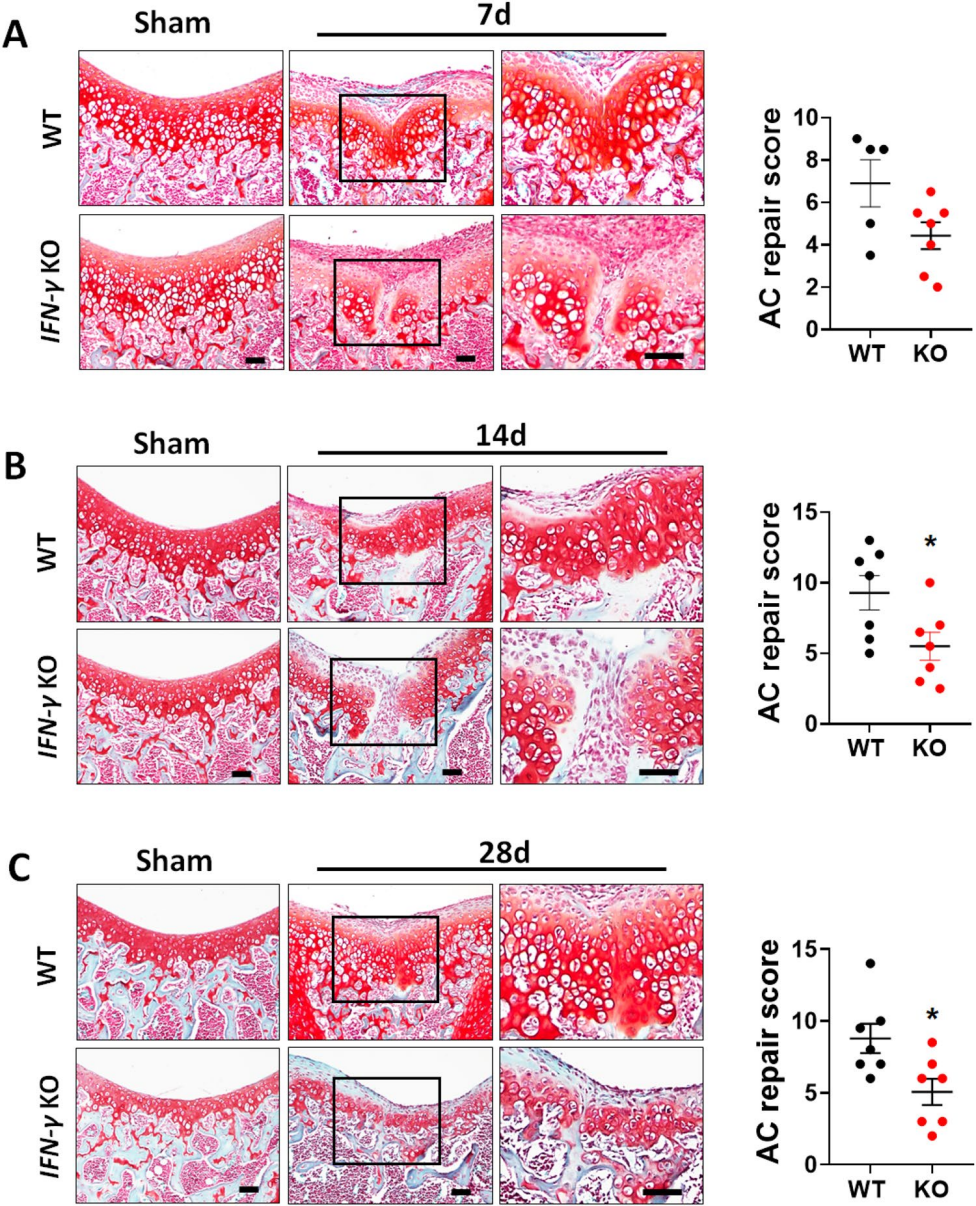
Genetic ablation of IFN-γ impairs cartilage regeneration after FTCI in 3-week-old mice. (**A**–**C**) Cartilage regeneration was visualized by safranin-O/fast green staining at 7 (**A**), 14 (**B**), and 28 days (**C**). Quantitative analyses of the articular cartilage repair scores at each time point were performed (n = 5–7 per group). Boxed areas are shown at higher magnification. Scale bar = 50 μm. Data are shown as mean ± SEM. *P < 0.05 versus WT controls (unpaired two-tailed *t*-test).

To further clarify the role of IFN-γ signaling in cartilage regeneration, we also examined whether genetic ablation of IFN-γ impairs cartilage regeneration. IFN-γ^−/−^ mice have been reported to develop normally in the absence of pathogen challenge^32^. These mice showed a significant decrease in cartilage thickness compared to WT mice at 5 weeks of age (Supplementary Fig. 6A). However, no differences in the expression of anabolic matrix genes between IFN-γ^−/−^ and WT mice were revealed by qRT-PCR (Supplementary Fig. 6B). Three-week-old WT and IFN-γ^−/−^ mice were subjected to FTCI and cartilage regeneration was assessed 7, 14, and 28 days after injury. Notably, IFN-γ^−/−^ mice displayed impaired cartilage regeneration at all time points and had significantly lower cartilage repair scores 14 and 28 days after injury compared to WT mice (Fig. 7A–C). There were no signs of infection in IFN-γ^−/−^ mice after FTCI.

### IFN-γR1 is downregulated in OA cartilage

Having shown that IFN-γR1 deficiency delays cartilage regeneration and considering that a lack of intrinsic repair after cartilage injury is a major contributor to OA, we examined whether a decrease in IFN-γR1 expression is associated with mouse or human OA. Using immunohistochemical (IHC) analysis, we quantified IFN-γR1 expression in OA-affected human cartilage obtained from individuals who had undergone arthroplasty, as well as in mouse cartilage in which OA was induced by destabilization of the medial meniscus (DMM). IFN-γR1 was abundant in normal human and sham mouse cartilage, whereas its expression was markedly decreased in human and mouse OA cartilage (Supplementary Fig. 7A,C). The proportion of IFN-γR1-positive cells and staining intensity were significantly decreased in both human and mouse OA cartilage (Supplementary Fig. 7B,D), suggesting that the expression of IFN-γR1 is negatively associated with OA pathogenesis.

### OA is exacerbated in IFN-γR1-deficient mice

**Figure 8:**
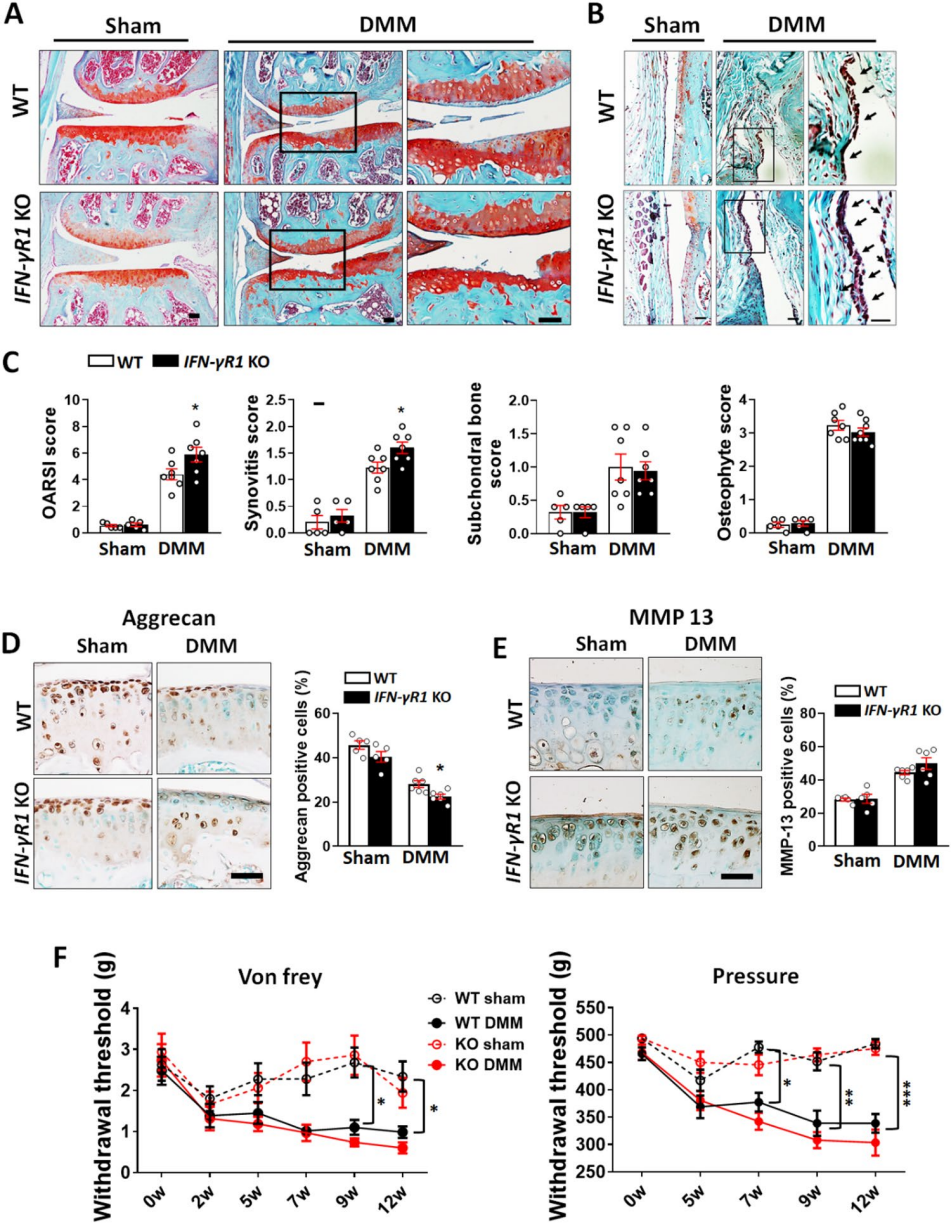
Genetic ablation of IFN-γR1 exacerbated OA pathogenesis in mice after DMM. (**A**) Representative images of safranin-O/fast green-stained sections of the knee joints of WT and IFN-γR1^−/−^ mice after DMM. Boxed areas in the right panel are shown at higher magnification. Scale bar = 50 μm. (**B**) Representative images of the synovium in the knee joint of each group. Boxed areas in the right panel are shown at higher magnification. Arrows indicate increased synovial cell proliferation. Scale bar = 50 μm. (**C**) Quantitative analyses of the Osteoarthritis Research Society International (OARSI), synovitis, subchondral bone, and osteophyte formation scores after DMM in WT and IFN-γR1^−/−^ mice (n = 5–7). (**D**,**E**) Representative images of immunohistochemical staining for aggrecan (**D**) and matrix metalloproteinase (MMP)-13 (**E**) expression in knee sections of WT and IFN-γR1^−/−^ mice after DMM. Quantification of aggrecan- and MMP-13-positive chondrocytes in the articular cartilage of sham-operated and DMM joints (n = 5–6 per group; five sections/mouse). Scale bar = 50 μm. (**F**) Mechanical allodynia was evaluated using the von Frey filament test (left; n = 8–10) and the withdrawal threshold was measured using a pressure algometer (right; n = 6–7) after DMM. Data are shown as mean ± SEM. *P < 0.05, **P < 0.01, ***P < 0.001 versus WT sham or WT DMM according to the two-tailed Mann–Whitney U test (**C**), unpaired two-tailed *t*-test (**D**,**E**), and Kruskal–Wallis test with Dunn’s multiple comparison test (**F**).

Next, we determined whether IFN-γR1 ablation exacerbates OA. Ten-week-old IFN-γR1^−/−^ and WT mice were subjected to DMM surgery. Twelve weeks after DMM, significantly more severe cartilage erosion in the medial tibial plateau was observed in IFN-γR1^−/−^ than in WT mice; the former group also had a significantly higher Osteoarthritis Research Society International (OARSI) score (Fig. 8A,C). In addition, IFN-γR1^−/−^ mice had higher levels of cell infiltration and a thicker synovium, indicating more severe synovial inflammation compared to WT mice (Fig. 8B,C). However, there were no significant group differences in subchondral bone sclerosis or osteophyte formation (Fig. 8C). IHC analysis revealed a decrease in aggrecan expression and an increase in matrix metalloproteinase (MMP) expression in the articular cartilage of IFN-γR1^−/−^ mice compared to WT mice after DMM (Fig. 8D,E). OA-associated pain was assessed after DMM. The WT and IFN-γR1^−/−^ mice showed similar pain responses, as measured using the von Frey filament test and a pressure algometer (Fig. 8F).

### Aged IFN-γR1-deficient mice exhibit a more severe OA-like phenotype

Finally, we assessed the contribution of IFN-γR1 to the development of naturally occurring OA in ageing (16-month-old) mice. WT mice displayed proteoglycan loss in the femoral condyle and tibial plateau, osteophyte formation, and subchondral bone thickness (Supplementary Fig. 8A). Notably, IFN-γR1^−/−^ mice tended to exhibit more severe OA phenotypes; they had higher OARSI, synovitis, subchondral bone, and osteophyte scores than WT mice, although the differences were not statistically significant (Supplementary Fig. 8B).

## Discussion

Because previous studies have reported that young mammals have greater cartilage regeneration capacity than adults, we investigated the mechanisms underlying the difference in cartilage self-regeneration capacity between young and adult mice. RNA-seq analysis of cartilage 3 days after FTCI revealed that immune pathway genes, particularly IFN signaling-related genes, were upregulated in young compared to adult mice. IFN-γ increased the proliferation and migration of chondrocytes, and IFN-γR1- and IFN-γ-deficient mice had less capacity for cartilage regeneration. In addition, DMM-induced and age-related OA were exacerbated in IFN-γR1 KO mice.

Accumulating evidence suggests that inflammatory cells and cytokines play integral roles in tissue regeneration. Moreover, IFNs may play a role in tissue regeneration by regulating the immune response. In this study, the expression levels of the antiinflammatory M2 markers arginase-1 and CD206 were significantly elevated, whereas the expression of the proinflammatory M1 marker CD16 was decreased, 3 days after cartilage injury in young compared to adult mice. By contrast, total macrophage accumulation (F4/80) was greater, and the expression of the proinflammatory mediators TNF-α and iNOS was increased in parallel with increases in IFN-γ and IFN-γR1 in regenerating tissue at 3 days after injury in young mice. This suggests that the cartilage repair process in young animals is accompanied by both pro- and antiinflammatory signals, where a fine balance between these two signals may be required for optimal cartilage repair. In a mouse model of cardiotoxin muscle injury, IFN-γ null mice showed impaired muscle healing, which was in turn associated with impaired macrophage function. Moreover, IFNGR-blocking antibodies reduced the proliferation and fusion of muscle cells^33^, suggesting an important role of endogenous IFN-γ signaling in tissue regeneration. By contrast, IFN-γ supplementation exacerbated muscle pathology in mdx mice, a Duchenne muscular dystrophy model, by promoting a cytolytic M1 and suppressing proregenerative M2 macrophages^34^. The discrepant results may be attributable to the use of different disease models (injury vs. genetic), as well as to differences in tissue characteristics and the source of IFN-γ (endogenous vs. exogenous). Our results suggest that the microenvironment of the site of injury can be altered in young mice, leading to rapid inflammatory cell infiltration and the scavenging of dead cells and cellular debris by resident macrophages.

Contradictory results have been reported regarding the role of IFN-γ in tissue regulation. IFN-γ has been reported to inhibit peripheral nerve- and bone marrow mesenchymal cell-based bone regeneration^35,36^. By contrast, IFN-γ-tethered hydrogels promoted tissue repair in a colonic mucosal wound model^37^, whereas IFN-γ blockade exacerbated skeletal muscle inflammation and fibrosis, and also impaired regeneration by inhibiting macrophage accumulation following muscle injury^38^. To the best of our knowledge, the role of IFN-γ signaling in cartilage regeneration has not previously been reported.

Low regenerative capacity of articular cartilage is a major challenge in the treatment of OA. We showed that IFN-γR1 expression is decreased in human and animal OA cartilage, suggesting a role of IFN-γ signaling in the cartilage degeneration characteristic of OA. Most previous studies that have investigated the influence of IFN-γ on chondrocytes and cartilage in vitro have observed catabolic responses, where IFN-γ decreased the viability and proliferation of chondrocytes, promoted the transcription of inflammatory mediators such as TNF-α, IL-6, and matrix-degrading enzymes, and inhibited the expression of aggrecan, decorin, and biglycan core protein in synergy with TNF-α^39–41^. However, a more recent study reported an anti-catabolic effect of IFN-γ on articular cartilage: IFN-γ downregulated MMP synthesis driven by IL-1β and attenuated glycosaminoglycan depletion in bovine articular cartilage co-cultured with synovium^42^. These results suggest that IFN-γ may have multi-faceted functions in the intraarticular microenvironment.

A recent report that compared the proteomic and secretome responses of fetal and adult ovine articular cartilage after injury showed that neutrophil-related and acute-phase proteins were upregulated in adult cartilage, whereas no significant change was observed in fetal cartilage despite a superior regenerative response^12^. IFN-related signals were not detected in adult or fetal cartilage. The difference between these results and those of our study may be attributable to differences in the array methodology, as well as the age and species of animals used in the experiments.

Our study had some limitations. First, we did not duplicate the RNA-seq results in another group of mice due to limited resources. However, most of the upregulated genes were validated by qRT-PCR in an independent set of experiments. Moreover, although we meticulously cleaned and isolated the articular cartilage of each animal, contamination of non-cartilage tissue was inevitable. However, in a chondrocyte-specific INF-γ KO animal model, the regenerative response was impaired, thus confirming the importance of cartilage IFN-γ signaling. Furthermore, we did not perform an experiment involving exogenous supplementation to further evaluate whether IFN-γ enhances cartilage regeneration or abrogates OA in animals. Considering the differences in endogenous and exogenous IFN-γ signaling in muscle injury models, a supplementation experiment might have provided different results. Thus, the detailed mechanisms underlying the effects of IFN-γ signaling pathway should be further examined to confirm its therapeutic potential; this will be the goal of our future studies. Although we demonstrated that IFN-γ promotes chondrocyte migration and proliferation, we did not assess its influence on other factors involved in the regeneration microenvironment, such as bone marrow mesenchymal stem cells and synoviocytes. Finally, although type 1 IFN in cartilage is differentially upregulated between young and adult mice, this was not evaluated in our study.

In conclusion, our study elucidates the functional role of IFN-γ signaling in cartilage regeneration. The results indicate that enhanced IFN-γ signaling could overcome the limited self-repair ability of cartilage and thus aid the treatment of OA.

### Supplementary Information

Supplementary Information.

## Data Availability

All processed data are available in the figures of the manuscript. All raw data that support the findings of this study are available from the corresponding authors upon request.
