# Supplementary material for: Interferon-gamma signaling promotes cartilage regeneration after injury
Source: Sci Rep. 2024 Apr 5;14:8046. doi: 10.1038/s41598-024-58779-0 (PMC10997668; doi:10.1038/s41598-024-58779-0)
Supplement: Supplementary file 1 — Supplementary Information. [file 41598_2024_58779_MOESM1_ESM.pdf]

Interferon-gamma signaling promotes cartilage regeneration after injury

Ju-Ryoung Kim, PhD[1,3,4], Bong-Ki Hong[3,4], Thi Hong Nhung Pham[1,2], Wan-Uk Kim[3,4], Hyun Ah Kim, MD, PhD[1,2]*

[1]Division of Rheumatology, Department of Internal Medicine, Hallym University Sacred Heart Hospital, Kyunggi, 14068, Korea, [2]Institute for Skeletal Aging, Hallym University, Gangwon-do 24252, Korea, [3]Division of Rheumatology, Department of Internal Medicine, School of Medicine, The Catholic University of Korea, Seoul 06591, Korea, [4]Center for Intergrative Rheumatoid Transcriptomics and Dynamics, School of Medicine, The Catholic University of Korea, Seoul 06591, Korea

***Correspondence to:**

Name: Hyun Ah Kim

Address: Division of Rheumatology, Department of Internal medicine, Hallym University Sacred Heart Hospital, 896, Pyungchon, Anyang, Kyunggi, 14068, Korea

Phone: 82-31-380-1826

Fax: 82-31-381-8812

E-mail: kimha@hallym.ac.kr

https://orcid.org/0000-0002-9318-7446

Materials and Methods

The cartilage repair, Osteoarthritis Research Society International (OARSI), synovitis, subchondral bone, and osteophyte scoring systems. All scoring were performed independently by two blinded observers. The cartilage repair scoring as follows[1], in the category of cell morphology, 4, hyaline cartilage; 3, mostly hyaline cartilage; 2, mostly fibro cartilage; 1, mostly non-cartilage; 0, non-cartilage only, in the category of matrix staining, normal; slightly reduced; markedly reduced, no staining, in the surface regulatory, smooth 100-75%; moderate 75-50%; irregular 50-25%; no staining, in the category of thickness of cartilage, >2/3 depth; 1/3-2/3 depth; <1/3 depth, in the integration with naïve cartilage, both edges integrated; one edge integrated; neither edge integrated. The OARSI scoring as follows[2], 0, normal; 0.5, loss of Safranin-O with no structural lesions; 1, small fibrillations with roughened articular surface; 2, vertical clefts down to the layer immediately below the superficial layer and some loss of surface lamina; 3, vertical clefts/erosion to the calcified cartilage extending to < 25% of the articular surface; 4, vertical clefts/erosion to the calcified cartilage extending to 25–50% of the articular surface; 5, vertical clefts/erosion to the calcified cartilage extending to 50–75% of the articular surface; and 6, vertical clefts/erosion to the calcified cartilage extending to > 75% of the articular surface. The synovitis score was carried out according to the two synovial membrane features including synovial lining cell layer and inflammatory infiltrate, ranking the changes being on a scale from none (0: one layer of lining cells and no inflammatory infiltrate), slight (1: 2-3 layers of lining cells and few mostly perivascular situated lymphocytes) and moderate (2: 4-5 layers of lining cells and numerous lymphocytes or plasma cells) to severe (3: the lining may be ulcerated, more than 5 layers and dense band-like inflammatory infiltrate)[3]. The subchondral bone was scored as follows[4], 0, less subchondral bone thickness than articular cartilage thickness in the load bearing area; 1, equal

subchondral bone thickness to articular cartilage thickness in the load bearing area; 2, greater subchondral bone thickness to articular cartilage thickness in the load bearing area. The osteophyte formation was scored as follows[5], 0, none; 1, formation of cartilage-like tissues; 2, increase in cartilaginous matrix; 3, endochondral ossification.

Table S1. Primer sequences for mouse genes analyzed by qRT-PCR

Gene	Primer sequence (5'-3')
GAPDH	Forward- CAT GGC CTT CCG TGT TCC
	Reverse- GCG GCA CGT CAG ATC CA
Aggrecan	Forward- GGA CAG GCT GGC TGA AGA
	Reverse- GAG ACC CCT GGG AAG GAA
Col2a1	Forward- GTG TGT GTG ACA CTG GGA ATG TC
	Reverse- GGT TGA GGC AGT CTG GGT CTT
Sox9	Forward- CGG CTC CAG CAA GAA CAA G
	Reverse- GCG CCC ACA CCA TGA AG
IFNGR1	Forward- TGT AGC CTC ACC GCC TAT CAC
	Reverse- GAT CCC ACG AGG CCA CTG T
IFNG	Forward- TTG GCT TTG CAG CTC TTC CT
	Reverse- TGA CTG TGC CGT GGC AGT A
Ccl6	Forward- CCA GTG GTG GGT GCA TCA A
	Reverse- GGG TTC CCC TCC TGC TGA TA
Slc11a1	Forward- CAG CTG TCA TGC AGG AGT TTG
	Reverse- TGC ACG AAG TGA TGG CTT TG
Ifitm3	Forward-GCC CCC AAA CTA CGA AAG AAT

	Reverse-GCG GTG CCC CCA TCT C
Mrc1	Forward-CCC AAG GGC TCT TCT AAA GCA
	Reverse-CGC CGG CAC CTA TCA CA
Bst2	Forward- TGG CGC CCT CTT TCT ATC AC
	Reverse- CCC CCC CAT CTC ATC CA
Ifitm6	Forward- GGC TGC TGC CTG GGT TT
	Reverse- TCT TCC GGT CCC TGG ACT T
Isg15	Forward- AAG AAG CAG ATT GCC CAG AA
	Reverse- TCG CTG CAG TTC TGT ACC AC
MyoD88	Forward-TCG ATG CCT TCA TCT GCT ATT G
	Reverse- CGG ATC ATC TCC TGC ACA AA
Stat1	Forward-CTC TGG AAT GAT GGG TGC ATT
	Reverse-TTG AGC AGA GCG CGT TCT C
Capg	Forward-GCC TCA AGT ACC GGG AAG GT
	Reverse-GCG CCC GAG GTT GTC TT
Trex1	Forward- ACT GCA GTG GGT GGA CGA A
	Reverse-TGC CGT ACA TGG GCT TGA C
Ifitm1	Forward-GCA GCA AGA GGT GGT TGT ACT G
	Reverse- TGG TGG CTG TCG CAG AAG T
Irf7	Forward- AAA TGC TGG GCT CCA AAC C
	Reverse- GAG GTC CCC GGC ATC AC
Vim	Forward-GAG AGA GGA AGC CGA AAG CA
	Reverse- GCC AGA GAA GCA TTG TCA ACA TC

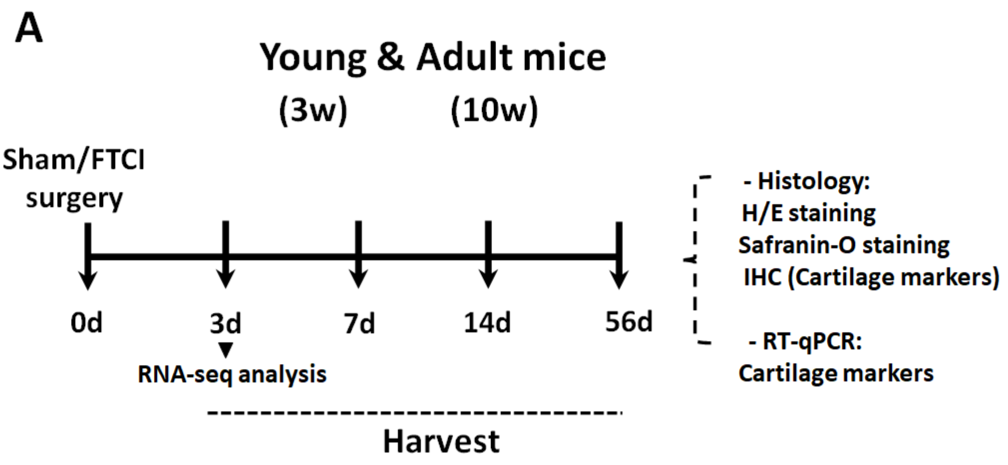

Supplementary figure 1. Experimental design for the induction of cartilage injury by full-thickness cartilage injury in young and adult mice. A, Three-week -old (young) or ten-week old (adult) male C57BL/6 mice were subjected to FTCI and assessment of cartilage regeneration was performed at 3, 7, 14, and 56 days post FTCI using a histological scoring system, proteoglycan staining, and chondrogenic marker staining and gene expression analysis.

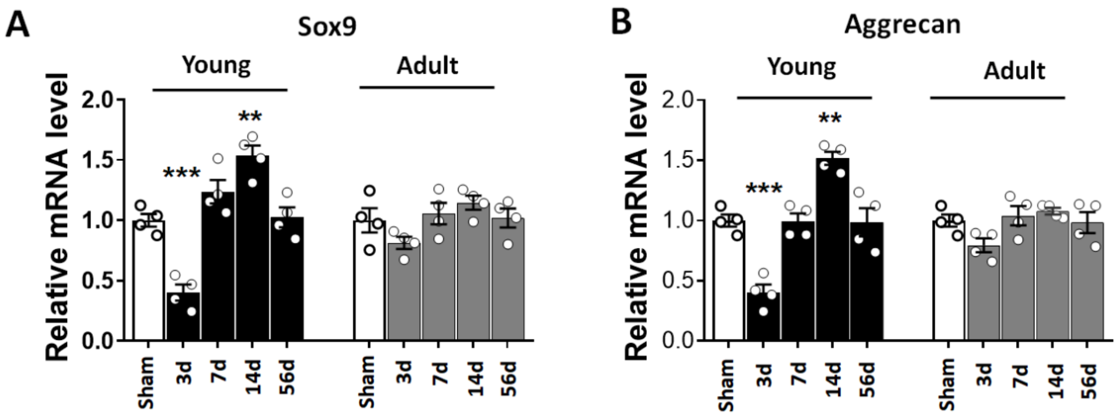

Supplementary figure 2. Expression of sox9 (A) and aggrecan (B) mRNA in the cartilage from young and adult mice after FTCI, as determined by real-time qRT-PCR. RNA was isolated from the cartilage following FTCI or from uninjured mice at each time point. For each group, cartilages were pooled from 2 sets of 10 mice before RNA extraction. The expression of glyceraldehyde-3-phosphate dehydrogenase (GAPDH) mRNA was used for normalization of data. Target gene expression are normalized by sham group at each time point and only one sham is represented as all shams have the same value at each time point. Data represent the fold increase in gene expression levels and shown as means ± SEMs of duplicate experiments from each set. $^{**}P < 0.01$, $^{***}P < 0.001$, $^{****}P < 0.0001$ vs. sham control group by one-way ANOVA with Tukey's multiple comparisons test.

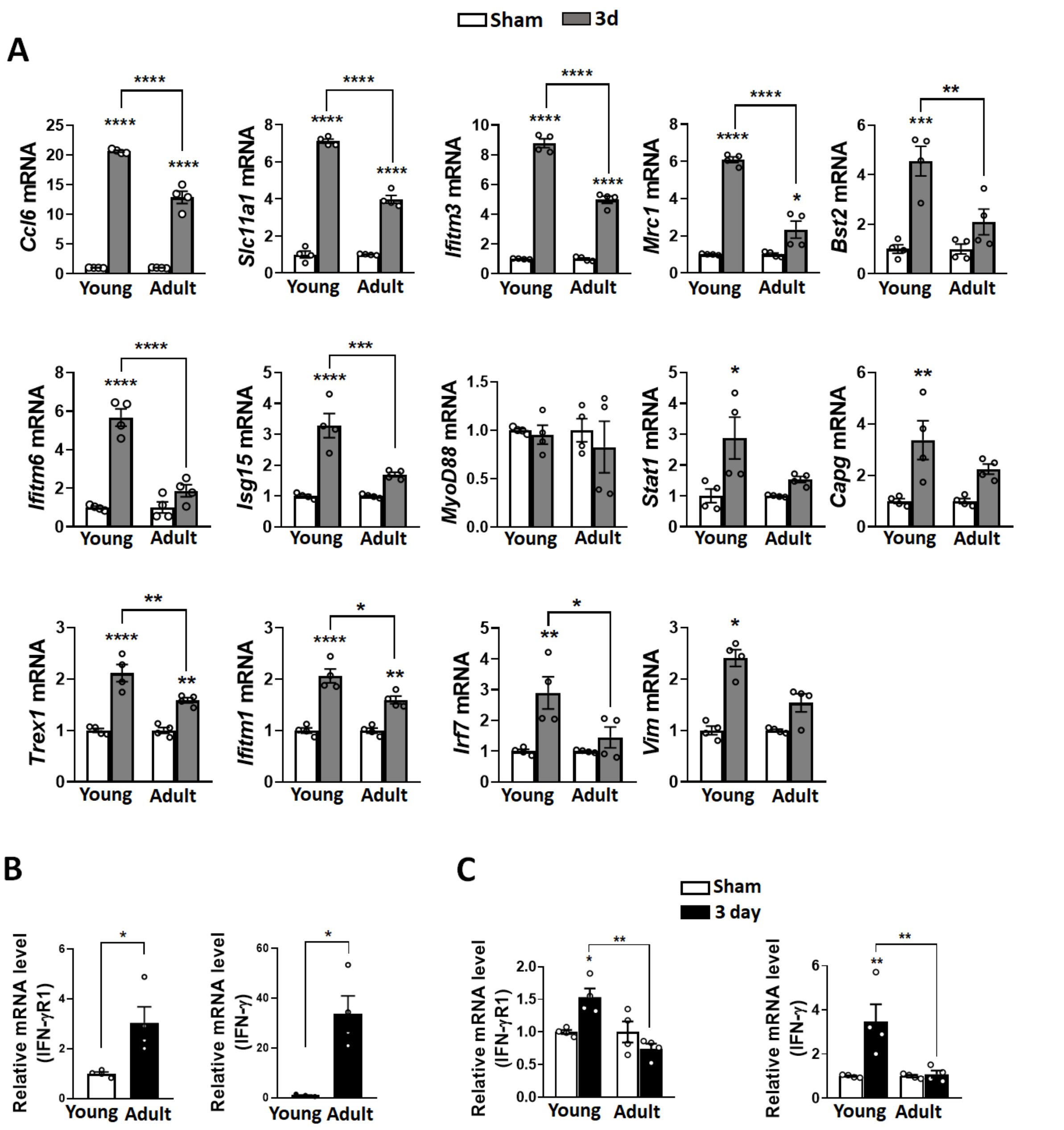

Supplementary figure 3. RT-qPCR validation of the expression of IFN-γ signaling pathway related genes from the RNA-seq data. For the validation, new RNA samples were extracted from a different set of cartilages at 3 days following FTCI or from sham mice. The injured femoral cartilages were pooled from at least 10 mice before RNA extraction and mRNA levels were analyzed by qRT-PCR. **A**, Expression levels of IFN-γ signaling pathway-associated genes. **B**, Expression levels of IFN-γR1 and IFN-γ mRNA in young and adult uninjured mice (n=10

for each group). **C**, Expression levels of mRNA IFN-γR1 and IFN-γ mRNA in young and adult mice after FTCI injury (n=10 for each group). Data are normalized to the expression of GAPDH mRNA and shown as means ± SEMs of duplicate experiments. *$P < 0.05$, **$P < 0.01$, ***$P < 0.001$, ****$P < 0.0001$ vs. sham control group. Statistical analyses were conducted using one-way ANOVA with Tukey's multiple comparisons test (A, all gens except for *Vim* and C) and Kruskal–Wallis test with Dunn's multiple comparisons test (A, for *Vim*) and unpaired two-tailed *t*-tests (B).

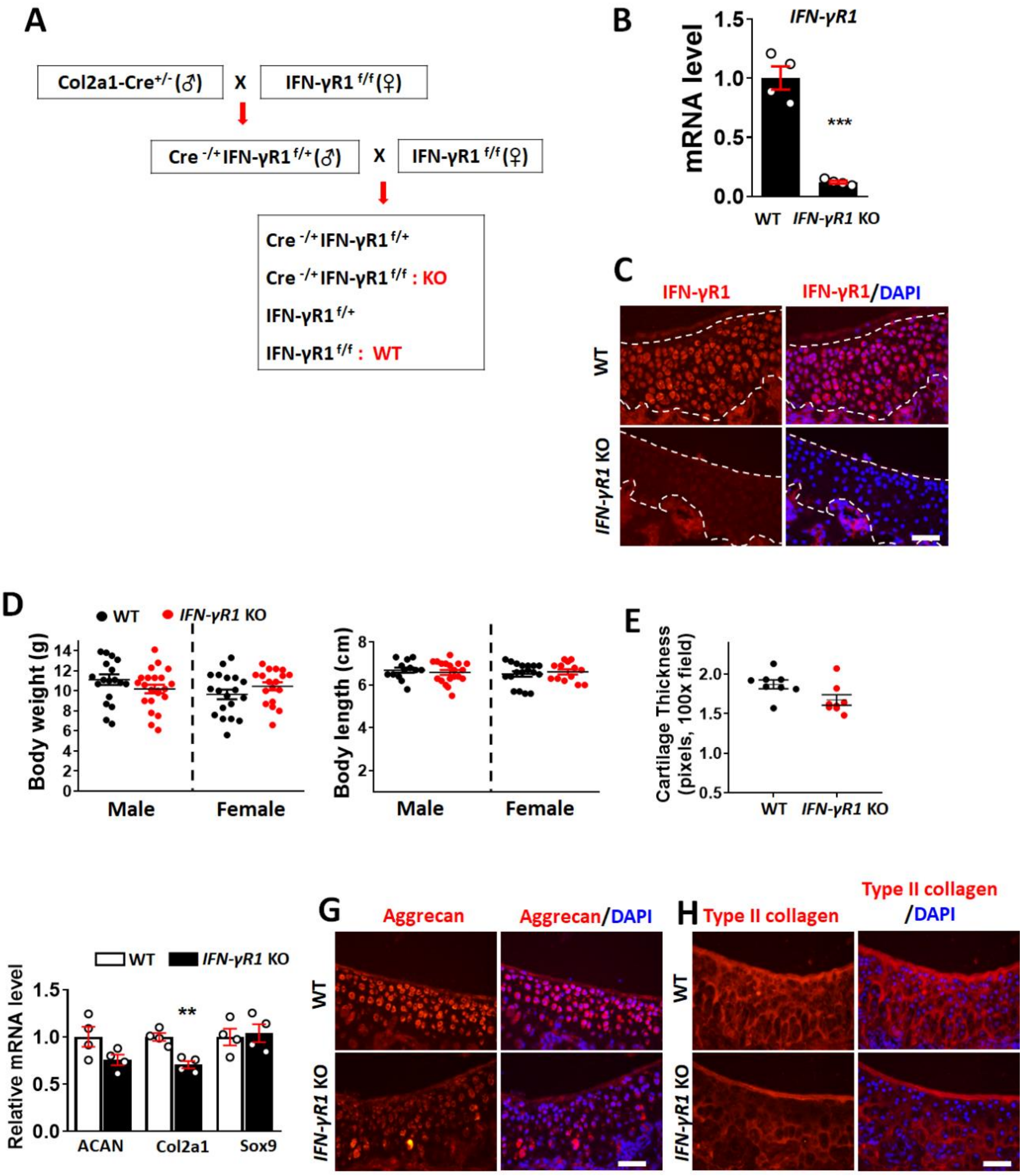

Supplementary figure 4. Generation of cartilage-specific IFN-γR1 knockout mice and characterization of the newborn littermates at 3-week-old **A**, Breeding scheme used to generate Col2a1- specific IFN-γR1 knockout mice. **B**, Quantitative analysis of IFN-γR1 transcript from pooled medial or lateral tibial and femoral cartilage of WT (n=10) and IFN-γR1$^{-/-}$ (n=10) mice by qRT-PCR. **C**, Representative image of immunofluorescence staining for IFN-γR1 in WT

and IFN-γR1$^{-/-}$ knee section. Scale bars=50 μm. Note the decrease in IFN-γR1 expression in the articular cartilage, but not in subchondral bone from IFN-γR1$^{-/-}$ mice. **D**, (Left) Body weight of WT (n=17 for male and n=19 for female) and IFN-γR1$^{-/-}$ (n=21 for male and n=18 for female) mice. (Right) Body length of WT (n=13 for male and n=18 for female) and IFN-γR1$^{-/-}$ (n=19 for male and n=13 for female). No difference was observed between genotypes or sex. **E**, Quantification of the articular cartilage thickness of the trochlear groove from WT and IFN-γR1$^{-/-}$ mice (n=7~8). Measurements from the subchondral bone to the articular cartilage surface were done at 3 points and averaged. Slight decrease in cartilage thickness was exhibited in IFN-γR1$^{-/-}$ mice. **F**, mRNA expression of aggrecan, Col2a1 and Sox9 from pooled medial or lateral tibial and femoral cartilage in WT and IFN-γR1$^{-/-}$ mice (n=10 for each group). **G, H**, Representative image of immunofluorescence staining for aggrecan (G) and Col2a1 (H) in WT and IFN-γR1$^{-/-}$ knee section. Scale bars=50 μm. Transcript data are normalized to the expression of GAPDH mRNA and shown as means ± SEMs of duplicate experiments. **P < 0.01, ***P < 0.001 vs. compared with WT control group according to unpaired two-tailed *t*-tests.

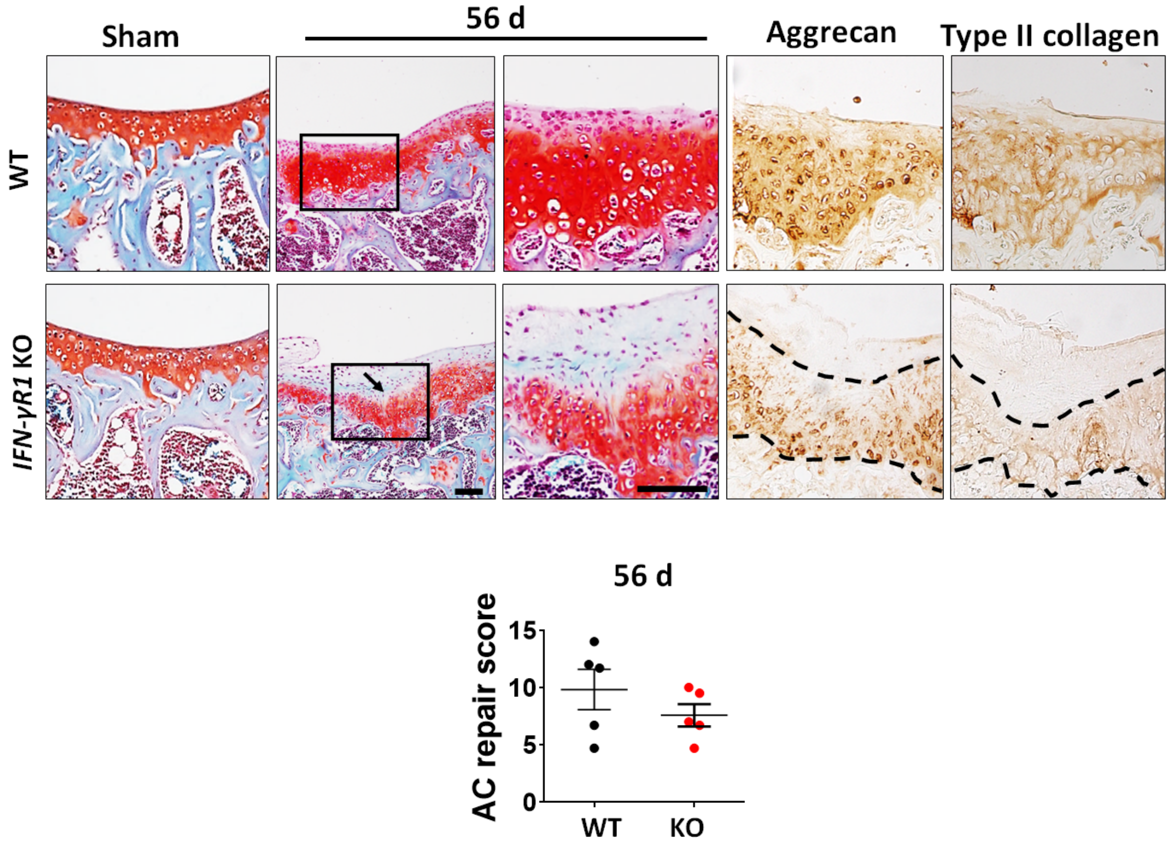

Supplementary figure 5. Genetic ablation of INF-γR1 delays cartilage regeneration after full thickness cartilage injury in 3-week-old mice**.** (Upper panels) Cartilage defect or regeneration was visualized by safranin-O/fast green staining at 56 days after injury in WT and IFN-γR1$^{-/-}$ mice. Boxed areas are shown at higher magnification. Complete hyaline cartilage was formed in WT mice, while IFN-γR1$^{-/-}$ mice exhibited fibrocartilage formation. Representative images of immunohistochemical staining for aggrecan and Col2a1 in the regenerating cartilage from WT and IFN-γR1$^{-/-}$ mice after injury. Arrows represent cartilage injury site. Dashed lines indicate cartilage part. Scale bars=50 μm. (Lower panels) Histological cartilage repair score was quantified (n=5 for each group, three sections/mouse). Data are shown as means ± SEMs.

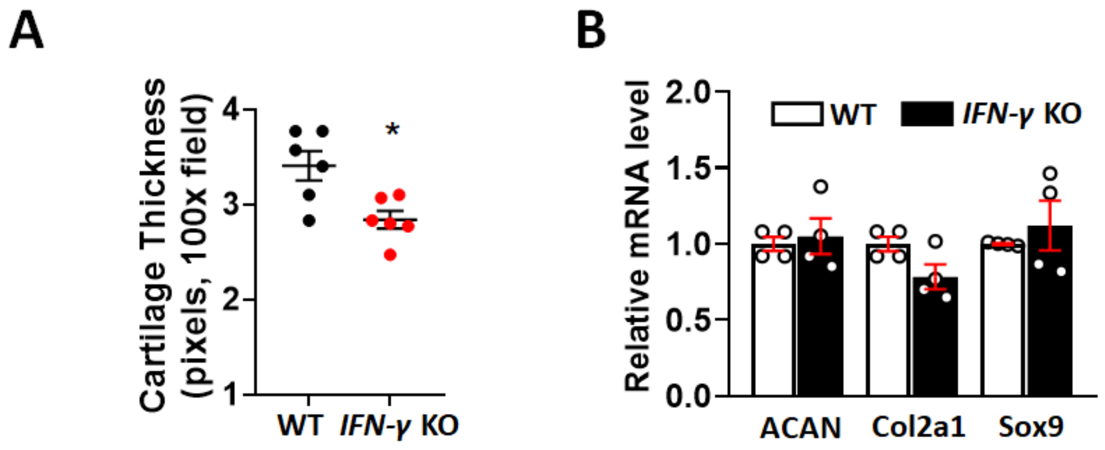

Supplementary figure 6. characterization of 5-week-old IFN-γ knockout mice. **A**, Quantification of the articular cartilage thickness of the trochlear groove from WT and IFN-$\gamma^{-/-}$ mice (n=6). Quantification of the articular cartilage thickness of the trochlear groove from WT and IFN-γR1$^{-/-}$ mice (n=6). Measurements from the subchondral bone to the articular cartilage surface were done at 3 points and averaged. Significant decrease in cartilage thickness was exhibited in IFN-$\gamma^{-/-}$ mice. **B**, Quantitative analysis of aggrecan, Col2a1 and Sox9 transcripts from pooled medial or lateral tibial and femoral cartilage in WT and IFN-γR1$^{-/-}$ mice (n=10 for each group) by qRT-PCR. Data are normalized to the expression of GAPDH mRNA and shown as means ± SEMs of duplicate experiments.

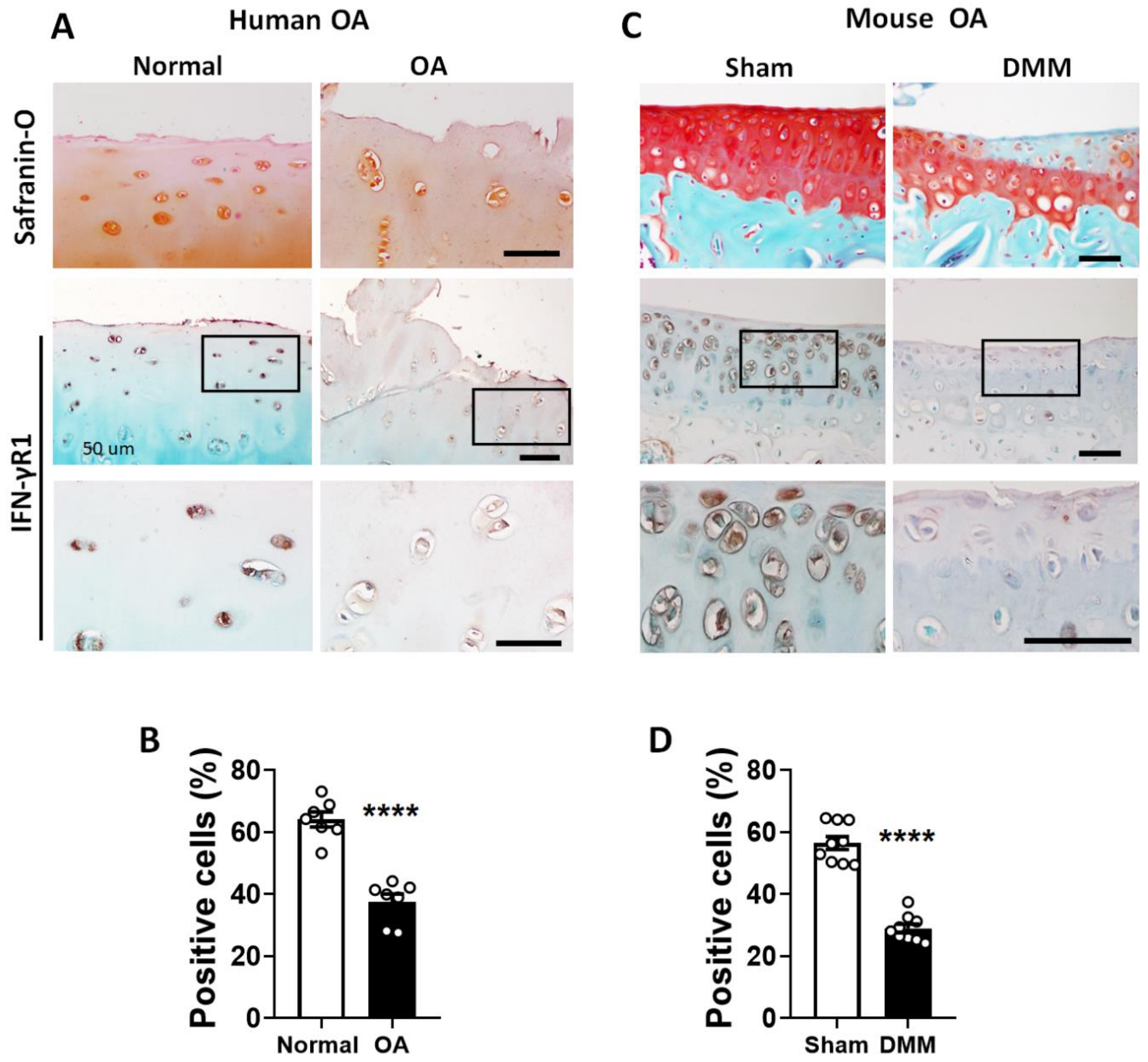

Supplementary figure 7. IFN-γR1 expression was downregulated in osteoarthritis (OA) cartilage. (A**)** Representative images of safranin-O staining (top) and immunostaining (bottom) for IFN-γR1 in normal and OA cartilage from human patients. (B) Quantification of IFN-γR1-positive chondrocytes as a proportion of total chondrocytes in normal and OA human articular cartilage tissues (normal cartilage, n = 7; OA cartilage, n = 7). (C) Representative images of safranin-O staining (top) and immunostaining (bottom) for IFN-γR1 in mouse cartilage after sham surgery or destabilization of the medial meniscus (DMM). (D) Quantification of IFN-γR1-positive chondrocytes in the articular cartilage of sham-operated and DMM joints (n = 5–

6 per group; seven sections/mouse). Scale bar = 50 µm. Data are shown as mean ± SEM. *P < 0.0001 versus the normal cartilage or sham control group (unpaired two-tailed *t*-test).

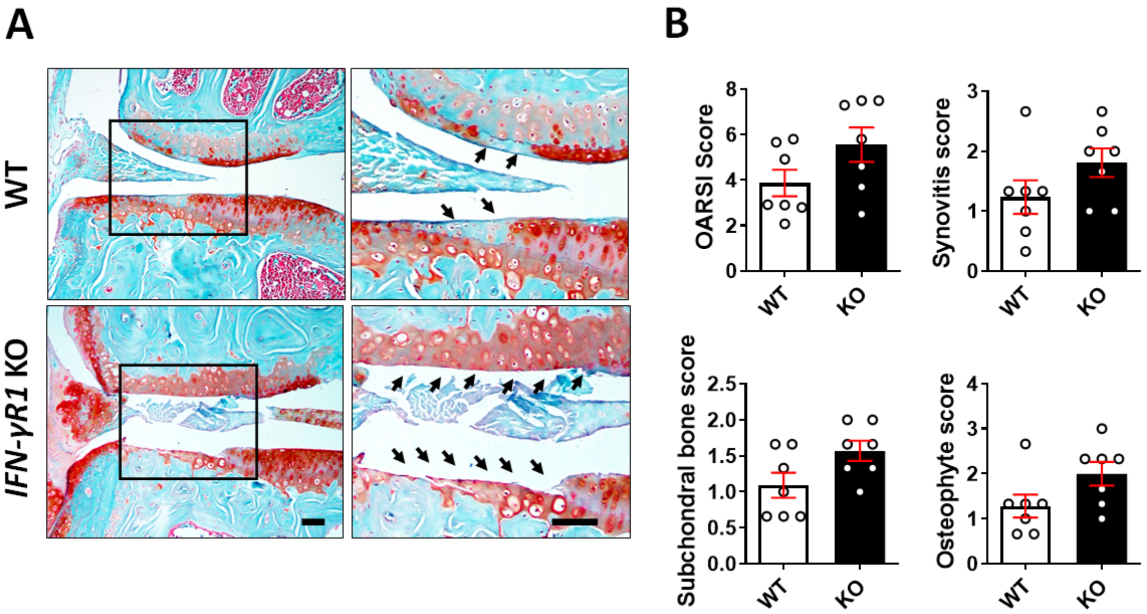

Supplementary figure 8. Aged IFN-γR1 deficient mice exhibit severer OA-like phenotype. **A**, Representative images of safranin-O/fast green stained sections of the knee joints of WT and IFN-γR1$^{-/-}$ mice at 16-month-old. Boxed areas are shown at higher magnification in the right panel. Arrows indicate areas of cartilage degeneration including proteoglycan loss and cartilage erosion. Scale bars = 50 μm. **B,** Quantitative analyses of the OARSI score, synovitis score, subchondral bone score, and osteophyte formation in WT and IFN-γR1$^{-/-}$ mice. (n = 7. 6~7 sections/mouse).

References

1 Matsuoka, M. *et al.* An Articular Cartilage Repair Model in Common C57Bl/6 Mice. *Tissue Eng Part C Methods* **21**, 767-772, doi:10.1089/ten.TEC.2014.0440 (2015).

2 Glasson, S. S., Chambers, M. G., Van Den Berg, W. B. & Little, C. B. The OARSI histopathology initiative - recommendations for histological assessments of osteoarthritis in the mouse. *Osteoarthritis Cartilage* **18 Suppl 3**, S17-23, doi:10.1016/j.joca.2010.05.025 (2010).

3 Krenn, V. *et al.* Synovitis score: discrimination between chronic low-grade and high-grade synovitis. *Histopathology* **49**, 358-364, doi:10.1111/j.1365-2559.2006.02508.x (2006).

4 Furman, B. D. *et al.* Joint degeneration following closed intraarticular fracture in the mouse knee: a model of posttraumatic arthritis. *J Orthop Res* **25**, 578-592, doi:10.1002/jor.20331 (2007).

5 Kamekura, S. *et al.* Osteoarthritis development in novel experimental mouse models induced by knee joint instability. *Osteoarthritis Cartilage* **13**, 632-641, doi:10.1016/j.joca.2005.03.004 (2005).
